# The Role of Peripheral Amide Groups as Hydrogen‐Bonding Directors in the Tubular Self‐Assembly of Dinucleobase Monomers

**DOI:** 10.1002/cplu.202100255

**Published:** 2021-06-29

**Authors:** Violeta Vázquez‐González, María J. Mayoral, Fátima Aparicio, Paula Martínez‐Arjona, David González‐Rodríguez

**Affiliations:** ^1^ Nanostructured Molecular Systems and Materials Group Organic Chemistry Department Science Faculty Universidad Autónoma de Madrid 28049 Madrid Spain; ^2^ Inorganic Chemistry Department Chemistry Faculty Universidad Complutense de Madrid 28040 Madrid Spain; ^3^ Institute for Advanced Research in Chemical Sciences (IAdChem) Universidad Autónoma de Madrid 28049 Madrid Spain

**Keywords:** cooperativity, nanotubes, self-assembly, supramolecular chemistry, supramolecular polymerization

## Abstract

Nanotubes are a fascinating kind of self‐assembled structure which have a wide interest and potential in supramolecular chemistry. We demonstrated that nanotubes of defined dimensions can be produced from dinucleobase monomers through two decoupled hierarchical cooperative processes: cyclotetramerization and supramolecular polymerization. Here we analyze the role of peripheral amide groups, which can form an array of hydrogen bonds along the tube axis, on this self‐assembly process. A combination of ^1^H NMR and CD spectroscopy techniques allowed us to analyze quantitatively the thermodynamics of each of these two processes separately. We found out that the presence of these amide directors is essential to guide the polymerization event and that their nature and number have a strong influence, not only on the stabilization of the stacks of macrocycles, but also on the supramolecular polymerization mechanism.

## Graphical abstract

**The supramolecular self‐assembly process** was compared for a set of guanine‐cytosine rod‐like dinucleobase monomers having a different number of peripheral amides, which revealed that these hydrogen‐bonding directing groups are essential to trigger and guide the stacking of Watson‐Crick‐bonded macrocycles into tubular nanostructures. These groups also have a strong influence on both nanotube stability, increasing with the number of peripheral amides, and on the polymerization mechanism, which can switch from cooperative to non‐cooperative.

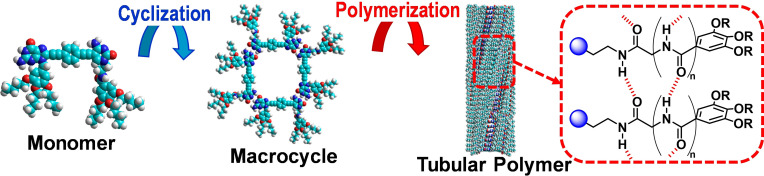

Chemists are increasingly motivated to design novel nanotubular systems, due to their wide diversity of functions,^[1]^ their nanometer‐scale dimensions, and their high aspect ratio. In fact, tube‐forming proteins, such as tubulin or aquaporin, or viruses like the tobacco mosaic virus, are seen as a truly amazing category of self‐assembled systems with biological functions. Possible applications of such materials with nanospaces of confined dimensions^[2]^ involve molecular sieve technologies,^[2b]^ chemo‐ and size‐selective encapsulation and storage, catalysis and sensing,^[2a]^ or biomedical applications, such as drug transport and biological ion channel mimics.[^2c^, ^2d^, ^2e^]

Probably the most important aspects in the design of nanotubes or nanotube networks are the modulation of their dimensions (i. e., adjusting the inner pore, tuning nanotube diameter), as well as ruling internal and external functionality. In this context, self‐assembled nanotubes and nanochannels built from organic molecules arise as promising materials that offer some distinct advantages with respect to carbon, inorganic or polymeric^[3]^ materials. These include low‐cost preparation under equilibrium conditions, synthetic versatility, biocompatibility, as well as precise tailoring of structure and function.[^3^, ^4^] Inspired by natural systems, the idea is to program molecules with the required chemical information so that they spontaneously assemble into well‐defined tubular structures *via* a combination of non‐covalent interactions and cooperative effects.^[5]^

In this context, we recently reported on the self‐assembly of dinucleobase rod‐like monomers like **GC1** (Figure 1a), comprising complementary G and C bases at the edges, into tubular structures^[6]^ in alkane solvents.^[6a]^ Remarkably, this assembly process occurred through two decoupled cooperative steps of different hierarchy (Figure 1b): 1) a cyclotetramerization process^[7]^ controlled by Watson‐Crick G : C pairing and enjoying a high chelate cooperativity,^[8]^ and 2) a supramolecular polymerization in which the macrocycles orderly stack to generate the final nanotubes through a nucleation‐elongation mechanism.

**Figure 1:**
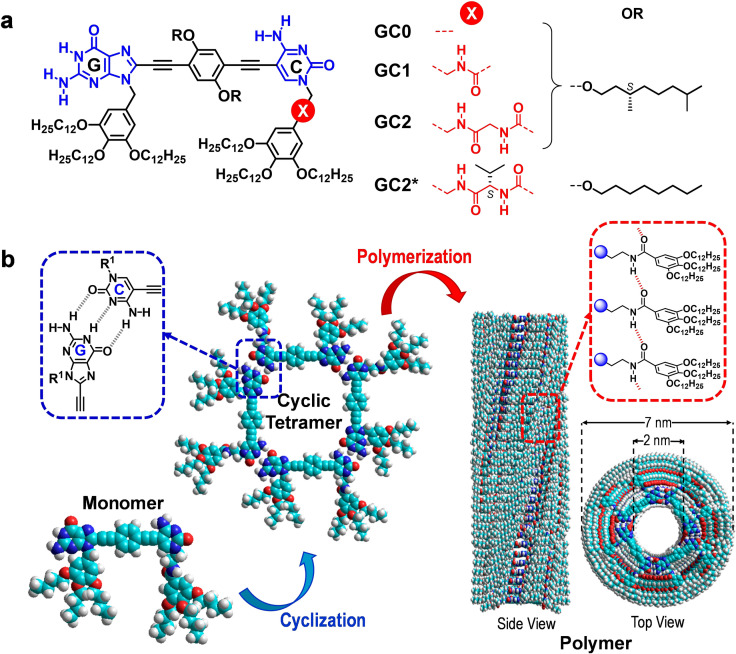
(**a**) Structure of *S*‐chiral **GC0**–**GC2** molecules (see also Figure 2). (**b**) Scheme of the two‐step self‐assembly of dinucleobase monomers.

The peripheral amide group placed at the C base in **GC1** was suspected to play a key role along the polymerization phase, stabilizing **GC1** tubular stacks by formation, just like in peptide β‐sheets, of an array of H‐bonds parallel to the tube's main axis, so we called them “parallel directors”. Here, we analyze the influence of these groups on this self‐assembly process. We prepared three other related monomers (Figure 1a) endowed with 0 (**GC0**) or 2 (**GC2** and GC2*) peripheral amides and studied their self‐organization, focusing on both the cyclotetramerization and the polymerization processes. We reveal that these parallel directors are indeed crucial to trigger polymerization, and that they greatly contribute to the stabilization of the tubular assemblies.

**Synthesis**. Monomers **GC0**–**GC2** are endowed with a *p‐*phenylene central block having lateral alkyl chains with *S*‐chiral centers, and thus only differ in the number of peripheral amide groups placed at the C unit. Monomer GC2* is analogous to **GC2**, but we moved the chiral center to the lateral chain at the C base by installing a valine derivative. However, despite showing a similar association behavior as **GC2**, evolving from a cyclic tetramer structure to longitudinal aggregates of the expected diameter (see below), the assemblies of GC2* turned out to be inactive in CD, an essential technique used in these studies, so we discarded this compound for subsequent experimental comparisons. The synthesis and molecular characterization of the cytosine precursors, as well as the novel dinucleobase monomers **GC0**, **GC2** and GC2* (Scheme 1), is detailed in the Experimental Section, as well as in the Supporting Information.

**Scheme 1:**
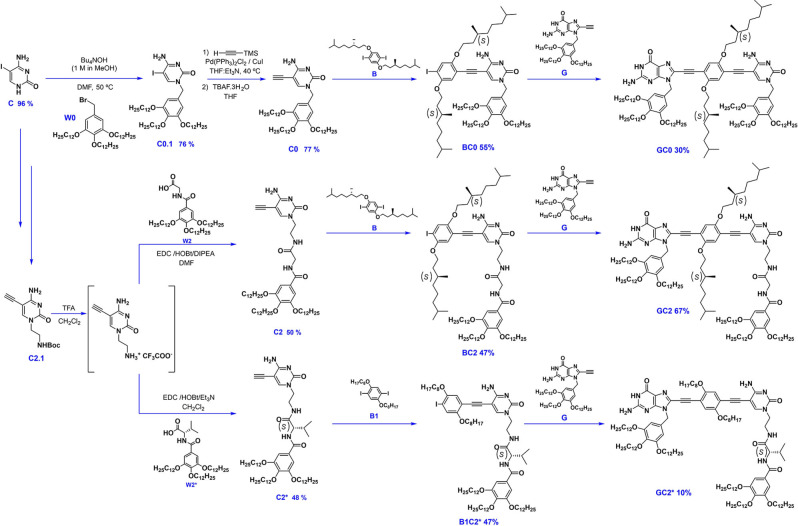
Synthesis of the new cytosine derivatives **C0**, **C2** and C2* and final monomers **GC0**, **GC2** and GC2*.

The synthesis of **GC1** and the rest of nucleobase and central block precursors can be found in our previous work.^[6a]^ Cytosine **C0** ^[9]^ was prepared from 5‐iodocytosine by an alkylation reaction with the benzylic wedge **W0**,^[10]^ followed by installation of the terminal acetylene *via* Sonogashira coupling with trimethylsilylacetylene (TMSA) and subsequent deprotection of the trimethylsilyl (TMS) group in the presence of fluoride ions. On the other hand, precursors **C2** and C2* were prepared from the amine‐protected cytosine **C2.1** ^[9]^ by Boc‐deprotection, and subsequent condensation reaction with acids **W2** ^[11]^ or W2*, respectively, whose preparation is further detailed at the S.I. Then, monomers **GC0**, **GC2** and GC2* were synthesized by two consecutive Sonogashira cross‐couplings between diiododerivatives **B** ^[12]^ or **B1** ^[13]^ and, firstly, the 5‐ethynylcytosines **C0**, **C2** and C2*, to yield the corresponding monocoupled products, and, subsequently, the 8‐ethynylguanine **G**,[^9^, ^14^] to arrive to the final monomers.

**The cyclotetramerization process**. As stated in the Introduction, when these GC dinucleobase molecules are gradually exposed to experimental conditions that favor their association by H‐bonding, a cyclotetramerization process, guided by G : C Watson‐Crick pairing interactions, is triggered first without actually observing oligo/polymerization through monomer stacking. Such monomer (GC) ‐ cyclotetramer (*c*(GC)_4_) equilibrium was studied by a number of spectroscopic techniques. From our experience^[6a]^ and preliminary studies (Figure S1), a solvent of intermediate polarity like THF is the best candidate to study the cyclotetramerization process, which can be principally done with ^1^H NMR, CD and emission spectroscopies. Since all macrocycles are formed from similar complementary G and C nucleobases, we expected that the association process of all monomers during this first equilibrium would be comparable, at least qualitatively.

Temperature‐ and concentration‐dependent ^1^H‐NMR experiments in THF‐d_8_ (Figures 2a–c and S2A, C) were first carried out in order to calculate the molar fraction of *c*(GC)_4_ species in diverse conditions and to evaluate the thermodynamic stability of each cyclic tetramer. In all cases, the G‐amide and C‐amine proton signals were detected at around 13.5 and 10.0 ppm (see Figures S2A, C), which is typical for H‐bonded G : C pairs. High temperatures and low concentrations favor dissociation into GC monomers while low temperatures and high concentrations obviously promote *c*(GC)_4_ formation. It is interesting to remark that, for all monomers, only these two species were detected in slow exchange in the NMR timescale, and no sign of small open oligomeric species was noticed, highlighting the strong *all‐or‐nothing* cooperativity of the cyclotetramerization process. CD experiments in the same conditions (Figures 2d–f and S2B, D) were in agreement with the NMR results. As described in previous work, CD spectroscopy turns out to be also very informative to assess the formation of cyclic species along our whole studies with dinucleoside monomers.[^6a^, ^8a^, ^8e^] This kind of molecules can switch between different conformations by rotation around the σ‐bonds in their rigid π‐framework, that dispose the nucleobase Watson‐Crick edges either at the same side or at opposite sides of the molecule. Upon cyclotetramerization, this degrees of freedom are lost and the Watson‐Crick faces at the edge nucleobases are blocked and forced to point to the same side. Such conformational “locking” might enhance the interaction between the chiral groups and the π‐conjugated backbone, so that the system becomes CD active. Hence, the CD trends recorded upon cyclization can be used to detect and analyze quantitatively the cyclotetramerization process in a complementary way and in a lower concentration range, because non‐cyclic or non‐associated species are CD‐inactive.^[8]^ The fixation of the planar *syn* conformation upon cyclotetramerization also explains the marked red‐shifts observed in the absorption and emission spectra (see Figure S1). So, in the same experimental conditions, the cyclotetramerization trends monitored by ^1^H NMR and CD as a function of temperature or concentration closely overlap, as shown in Figures 2g and S2F.

**Figure 2:**
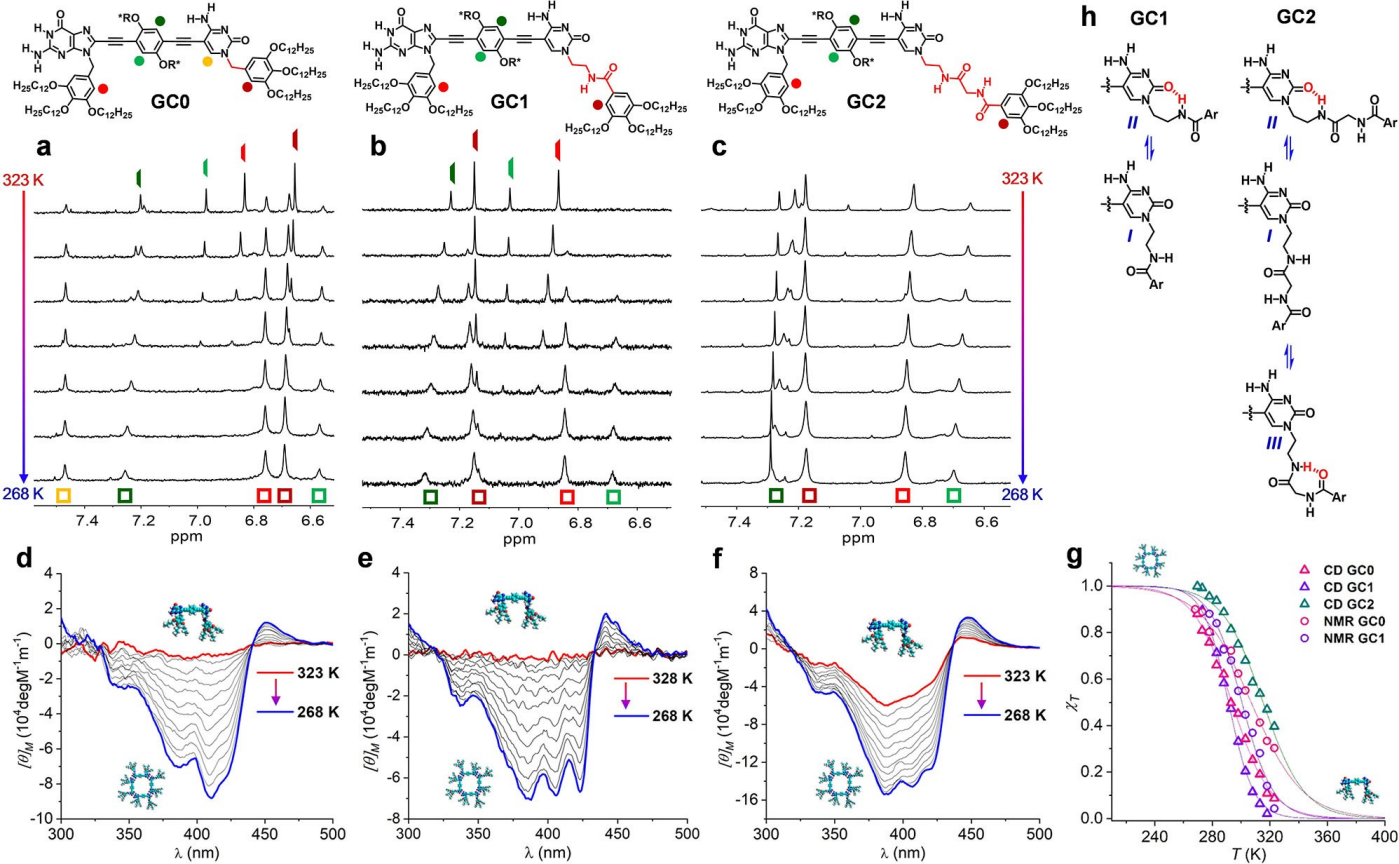
(**a**–**c**) Chemical structure and aromatic region of the ^1^H NMR spectra in THF‐d_8_ and (**d**–**f**) CD spectra in THF of compounds (**a**, **d**) **GC0**, (**b**, **e**) **GC1**, and (**c**, **f**) **GC2** as a function of the temperature. In all cases *C*=1.0 ⋅ 10^−4^ M. *c*(GC)_4_ and GC proton signals are marked with colored squares or rods, respectively. (**g**) Representation of the molar fraction of molecules associated as c(GC)_4_ cycles (*χ*_T_) calculated by ^1^H NMR (circles) or CD (triangles) as a function of the temperature. (**h**) Possible conformations adopted by the amide substituents at the C base. In some of them an intramolecular H‐bond may be formed leading to 7‐ (*II*) or 6‐membered (*III*) rings.

However, each macrocycle displayed a markedly different stability: *c*(**GC2**)_4_>*c*(**GC0**)_4_>*c*(**GC1**)_4_. For instance, at 298 K and at an identical 10^−4^ M concentration in THF, the molar fraction of molecules associated as cyclic tetramers (*χ*_T_) was >0.9 for **GC2**, 0.75 for **GC0** and 0.55 for **GC1**. These *χ*_T_ values as a function of concentration and temperature allowed us to calculate the cyclotetramerization constant (*K*_T_) and, through van't Hoff plots (Figure S2G), the corresponding enthalpy (Δ*H*) and entropy (Δ*S*) changes associated to the cyclotetramerization equilibria, which are compared in Table 1a. The only exception was **GC2**, for which, due to the strong association in these conditions, only a lower *K*_T_ limit could be determined.

**Table 1:** Thermodynamic parameters calculated for **GC0**–**GC2** upon (**a**) cyclotetramerization and (**b**) polymerization as a function of temperature.

**a**		***K***_G : C_^[a]^ [M^−1^]	***K***_**T**_^[b]^ [M^−3^]	***EM***^[c]^ [M]	Δ***H***_**T**_^[d]^ [kJ ⋅ mol^−1^]	Δ***S***_**T**_^[e]^ [J ⋅ mol^−1^ ⋅ K^−1^]
**GC0**		0.55 ⋅ 10^3^	4.3 ⋅ 10^13^	4.7 ⋅ 10^2^	−143.4	−215.1
**GC1**		0.41 ⋅ 10^3^	3.4 ⋅ 10^12^	1.2 ⋅ 10^2^	−173.4	−331.2
**GC2**		0.77 ⋅ 10^3^	> ⋅ 10^14^	>2.8 ⋅ 10^2^	^[f]^	^[f]^
**b**	***T***_**e**_^[g]^ [K]	***K***_**n**_^[h]^ [M^−1^]	***K***_**e**_^[i]^ [M^−1^]	***σ*** ^[j]^	Δ***H*** ^0[k]^ [kJ ⋅ mol^−1^]	Δ***S*** ^0[l]^ [J ⋅ mol^−1^ ⋅ K^−1^]
**GC1**	298	4.0 ⋅ 10^1^	1.3 ⋅ 10^5^	3.0 ⋅ 10^−4^	−116±3	−290±10
	***T***_**m**_^[m]^ [K]	***K*** [M^−1^]			Δ***H*** ^**0**[k,n]^ [kJ ⋅ mol^−1^]	Δ***S*** ^**0**[k,n]^ [J ⋅ mol^−1^ ⋅ K^−1^]
**GC2**	279	4.0 ⋅ 10^4^		1	−83±2	−192±6

[a] Reference G : C association constant calculated in separate experiments (see Figures 3 and S3A). [b] Cyclotetramerization constant. [c] Effective molarity calculated as: *EM*=*K*_T_/*K*_G : C_^4^. [d] Cyclotetramerization enthalpy and [e] entropy. [f] Could not be determined. [g] Elongation temperature at 3.0 ⋅ 10^−5^ M. [h] Nucleation and [i] elongation constants, [j] cooperativity factor, and elongation [k] enthalpy and [l] entropy of the polymerization process. [m] Melting temperature calculated from the isodesmic model. [n] Calculated using the van't Hoff equation.

In order to compare the cooperativity of each cyclotetramerization process, the corresponding reference association constants for each G : C nucleobase pair (*K*_G : C_)^[15]^ were measured in separate titration experiments (see Figure 3 and S3 A), and the effective molarity (*EM*) was calculated using the relationship *K*_T_=*EM* ⋅ *K*_G : C_^4^. Thus the noted differences in cycle stability may either come from a different *EM* for each cycle, from the subtle differences in G : C association constants that the lateral substituents at the C nucleobase may cause, or from a contribution of both parameters. From the data shown in Table 1a, it is clear that, even if there are not important differences in *K*_G : C_, the fact that each cycle is bound by 4 G : C interactions, and thereof *K*_G : C_ is raised to the 4^th^ power in the equation, can already explain the notable differences in cycle stability. The calculated *EM* values are, on the other hand and as expected, very similar for each cycle and in the order of 10^2^ M, thus in the same range as those calculated for related cycles made from G−C dinucleosides.^[8a]^

**Figure 3:**
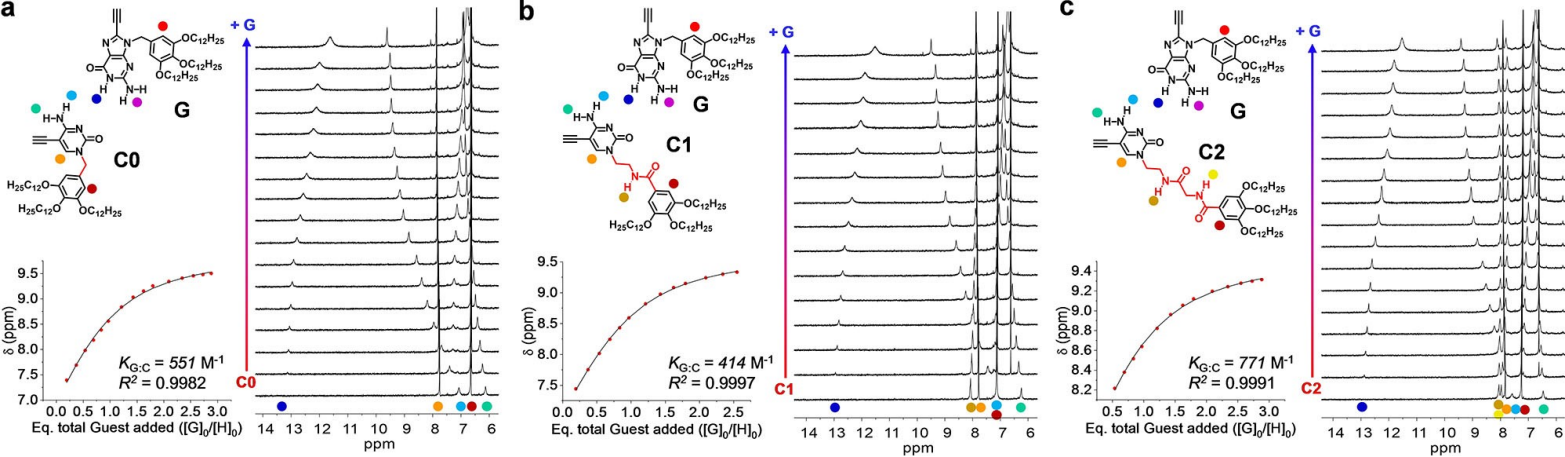
^1^H NMR titration experiments of **G** (guest) over (**a**) **C0**, (**b**) **C1**, or (**c**) **C2** (host) at a constant host concentration of 5 mM in THF‐d_8_. The corresponding fittings to a 1 : 1 model are shown at the left.

**The polymerization process**. We next investigated the supramolecular polymerization to yield self‐assembled nanotubes. This is a second stage in the overall self‐assembly landscape of these GC dinucleobase monomers that is only triggered when the molecules are exposed to experimental conditions that really increase their degree of aggregation, like in highly apolar solvents. In such conditions, as was demonstrated for **GC1** in our previous work,^[6a]^ and as will be proven below for the rest of GC molecules studied in this work, the cyclic tetramers are already formed quantitatively, meaning that both processes (i. e., cyclotetramerization and supramolecular polymerization) are totally decoupled. The number of peripheral amides is expected to play an important role in this polymerization process, since they should be involved in enhancing stacking interactions between the macrocycles, thus contributing to the stabilization of the resulting nanotubes. As also shown in our previous work,^[6a]^ both the cyclotetramerization and polymerization processes can be monitored by CD, absorption or emission spectroscopy. When analyzing and comparing the trends taken from each technique, they perfectly overlap at any concentration and provide virtually the same thermodynamic data. However, CD is the technique that provides a higher difference between cyclotetramer and polymer spectroscopic features, both visually and for quantitative analyses.

We first monitored the spectroscopic changes occurring upon increasing the volume fraction of heptane (*V*_h_) in mixtures with THF, from *V*_h_=0 to *V*_h_=0.99, at different concentrations (Figures 4a–c and S4A–C). As shown in Figure 4d, two processes were observed for **GC1** and **GC2** upon increasing *V*_h_: the monomer‐macrocycle equilibrium was monitored from *V*_h_=0 to *ca*. 0.4‐0.5. At that point a CD spectrum that is virtually identical for all molecules is obtained, with a strong negative signal and a weak, positive Cotton effect with zero‐crossing at *ca*. 439 nm. Then, a plateau is reached up to *V*_h_=0.7–0.8 in which this CD spectrum remains invariable, meaning that the *c*(GC)_4_ tetramers are the only species in solution. The supramolecular polymerization of the tetramers was then activated at higher heptane contents. This is experimentally observed by a drastic change in the CD spectra, and the stacked tubular polymers now show a strong bisignated signal with zero‐crossing at *ca*. 428 nm. Interestingly, the polymerization of c(**GC2**)_4_, with two peripheral amides per monomer, took place at a significantly lower bad solvent content (*V*_h_=0.7) than the polymerization of *c*(**GC1**)_4_ (*V*_h_=0.85), which is endowed with a single amide per monomer. On the contrary, for **GC0**, devoid of these peripheral H‐bonding groups, only the cyclotetramerization process was recorded, and this compound remained associated as *c*(**GC0**)_4_ cyclic tetramers even in pure heptane. These findings indicated that the presence and number of peripheral amides plays indeed a key role in the formation of the polymeric nanotubes. At this point, it is interesting to note that GC2* shows a polymerization process somewhat less favourable than **GC2**, most likely as a result of a higher steric hindrance caused by the isopropyl group around the amide director. Although we could not record GC2*’s CD changes, we could compare the supramolecular behaviour of this molecule with the one shown by **GC2** in denaturalization experiments by increasing the volume fraction of THF in heptane at a constant temperature and concentration, monitored by absorption spectroscopy (Figure S4F).

**Figure 4:**
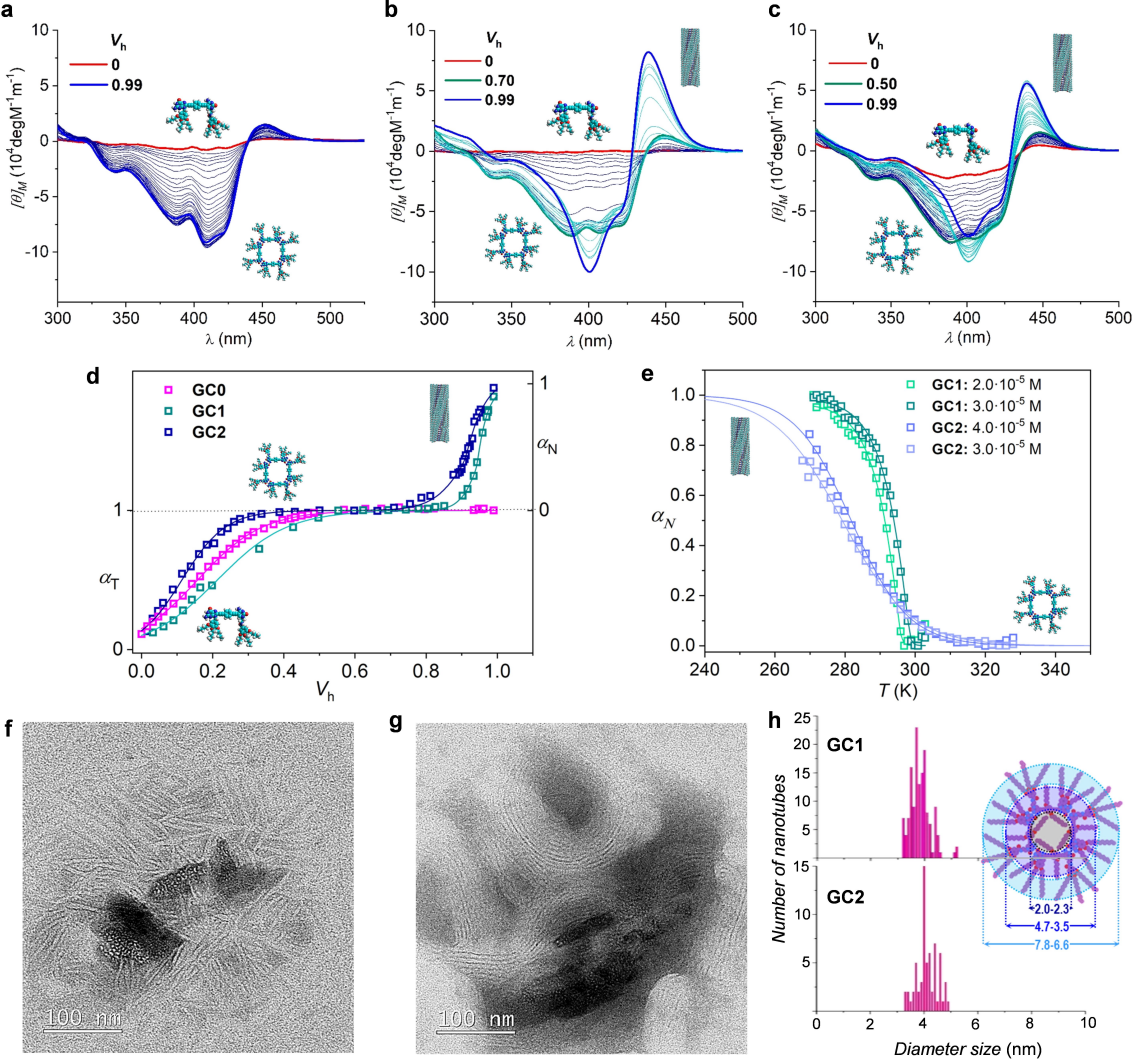
(**a**–**c**) Changes observed in the CD spectra as a function of the volume fraction of heptane (*V*_h_) in mixtures with THF for (**a**) **GC0**, (**b**) **GC1**, and (**c**) **GC2** (*C*=3.0 ⋅ 10^−5^ M; *T*=298 K). Normalized CD changes at 429 nm as a function of (**d**) *V*_h_ for **GC0**‐**GC2** or (**e**) the temperature for **GC1** and **GC2**. (*α_T_* =fraction of cyclotetramers, *α_N_* =fraction of aggregated nanotubes). (**f,g**) TEM images of the assemblies formed by **GC2** drop‐casted from diluted solutions of high *V*_a_. (**h**) Nanotube diameter distribution measured by TEM and dimensions calculated from models.

In order to have a deeper insight into the polymerization of *c*(**GC1**)_4_ and *c*(**GC2**)_4_, we conducted temperature‐dependent measurements monitored by CD and absorption at different concentrations and at a fixed solvent composition, which was chosen in each case so as to cover as much of the polymerization process as possible within the experimental temperature range (*V*_h_=0.97 for **GC1** and *V*_h_=0.81 for **GC2**; Figures 4e and S4E). As expected, the polymerization process is dependent on concentration and the elongation temperature decreases as the sample is concentrated and with the number of peripheral amides, indicating again a strongest association for **GC2** than for **GC1**. But surprisingly, the shape and the underlying analysis of the cooling curves revealed drastically different polymerization mechanisms. **GC1** exhibited a cooperative polymerization that was analyzed by a nucleation–elongation model,^[16]^ which allowed us to calculate the most important thermodynamic parameters (see Tables 1b and S2),^[6a]^ and revealed a small cooperativity factor (*σ*). On the contrary, **GC2** (and GC2*, see Figure S4F) displayed sigmoidal features that were analyzed by the isodesmic model, with σ=1.^[17]^

Although we do not have a clear explanation, we believe this difference is more likely due to the nature of the peripheral C substituents than to the fact that each cooling curve was measured at a different *V*_h_. As shown in Figure 2h, the pendant groups at the C base can adopt different conformations. In some of them the amide groups are bound to solvent molecules and in some others intramolecular H‐bonds can be established.^[18]^ We discard the formation of intermolecular H‐bonds between these amides at high THF contents, since their chemical shifts do not change with concentration (Figure S2C). A quite probable conformation for **GC1** and **GC2**, where a 7‐membered H‐bonded ring is formed intramolecularly, is represented in Figure 2h as conformation *II*. However, upon Watson‐Crick pairing, such conformation must become much less populated, because the C‐carbonyl group needs to bind the G base as well. Hence, cyclization would “release” such amide group, which would be available for polymerization. However, in **GC2**, a different kind of intermolecular H‐bond leading to a 6‐membered ring can be expected (conformation *III*), which might “sequester” the amide group and thus influence the polymerization mechanism.

We made a number of experiments to try to get a deeper insight into this issue without much success. On one hand, ^1^H NMR spectra down to 178 K were acquired with the individual C nucleobases (**C1** and **C2**; see Figure S3B), to try to “freeze” one of these conformations and detect it in slow exchange. However, the compounds precipitated below 218 K and the amide signals (and actually all protons involved in H‐bonding interactions) only showed a gradual downfield shift with decreasing temperature (see also Figure S2A). On the other hand, we recorded different ^1^H NMR spectra at gradually increasing volume fractions of cyclohexane‐d_12_ into THF‐d_8_, with the aim to see a change in the amide chemical shifts just when polymerization is triggered. However, when *c*(**GC1**)_4_ and *c*(**GC2**)_4_ start to polymerize, their ^1^H NMR signals become too broad and disappear before any H‐bonding rearrangement can be detected (Figure S2E). FT‐IR spectroscopy experiments performed in THF or THF:heptane (1 : 99) solutions or in the solid state (see Section S0 at the Supporting Information) did not help in providing further information either about the nature of the most abundant conformation in each of these situations. We analyzed both the amine/amide N−H region between 3000 and 3600 cm^−1^ and the carbonyl region between 1500 and 1800 cm^−1^. However, the high number of functional groups present in each monomer makes this analysis extremely difficult. For instance, a monomer like **GC2** has 4 C=O groups, 2 exocyclic NH_2_ groups and 3 N−H amide groups. Besides, depending on the conditions, some of these groups may be H‐bonded intramolecularly, intermolecularly, or some may be bound to solvent molecules, while some other may not be H‐bonded. We tried to simplify the system by comparing first the nucleobase fragments **C0**, **C1**, **C2** and **G**, but even there, no final conclusion could be obtained.

Finally, we drop‐casted **GC1, GC2** and GC2* samples from diluted solutions with *V*_h_ >0.8, and analyzed them by TEM. As Figures 4f‐h and S5 demonstrate, longitudinal objects with a measured diameter of 3.9±0.7 nm (**GC1**) or 4.0±0.8 nm (**GC2**), which coincides with the hard aromatic section of the cyclic tetramers, were imaged. We also noted that it is quite frequent to observe aligned nanotube bundles, especially as the time in solution before deposition becomes longer, which would be the result of van der Waals interactions between the peripheral alkyl chains of individual nanotubes. As a matter of fact, in some cases, especially if the sample concentration is high, we observed nanotube precipitation with time at the highest *V*_h_ values. It is important to note that SAXS and DLS measurements, as shown in our previous work for **GC1**,^[6a]^ were in agreement with the formation of large cylindrical aggregates with a cylinder diameter of 4±1 nm and a core diameter of about 1 nm.

In this work we focused on the analysis of the influence of the nature of the directors placed at the C base on the thermodynamics of the supramolecular processes to build self‐assembled nanotubes. We focused separately on the cyclotetramerization and the supramolecular polymerization events. We noted that these substituents lead to slight deviations in the association constants between G and C nucleobases that can however have a strong impact on macrocycle stability. More importantly, the presence of these amide groups is essential to trigger and guide the stacking of the *c*(GC)_4_ “supramonomers”, while their number greatly influences tube stability. Unexpectedly, the supramolecular polymerization mechanism is also modulated by these external groups, probably through a competition between intra‐ and intermolecular H‐bonding equilibria.

## Experimental Section

**General Methods**: Mass Spectrometry (MS) and High Resolution‐Mass Spectrometry (HRMS) MALDI‐TOF spectra were obtained from a BRUKER ULTRAFLEX III instrument equipped with a nitrogen laser operating at 337 nm. NMR spectra were recorded with a *BRUKER AVANCE‐II* 300 MHz or a *BRUKER DRX* 500 MHz instrument. The temperature was actively controlled at 298 K. Chemical shifts are measured in ppm using the signals of the deuterated solvent as the internal standard [CDCl_3_ calibrated at 7.26 ppm (^1^H) and 75.0 ppm (^13^C), DMSO‐d_6_ calibrated at 2.50 ppm (^1^H) and 39.5 ppm (^13^C) and THF‐d_8_ calibrated at 3.58 (^1^H) and 39.5 ppm (^13^C)]. Due to solubility problems the ^13^C‐NMR data for some final monomers and their derivatives could not be performed. Column chromatography was carried out on silica gel *Merck‐60* (230–400 mesh, 60 Å), and TLC on aluminium sheets precoated with silica gel 60 F254 (Merck). FT‐IR spectra were recorded with a *PerkinElmer* spectrometer UATR two. UV‐Visible experiments were conducted using a *JASCO V‐660* apparatus. Emission spectra were recorded in a *JASCO FP‐8600* equipment using excitation and emission bandwidths of 5 nm in both cases, and a 50 ms response. CD spectra were recorded with a *JASCO* J‐815 equipment. The slit width was set at 1000 μm and a DIT of 2 s was used. In all these three instruments the temperature was controlled using a *JASCO* Peltier thermostatted cell holder with a range of 263–383 K, adjustable temperature slope, and accuracy of ±0.1 K. Transmission electron microscopy (TEM) images were obtained with a *JEOL‐JEM 1010* instrument operating at 100 kV for the stained samples and a *JEM 1400K PLUS* instrument operating at 100 kV for non‐stained samples.

### Standard procedures

*Standard Procedure A* for the nucleobase alkylation reaction. To a suspension of the nucleobase starting material (1 eq) and a base (1.2 q) (indicated in each case) in dry DMF (volume indicated in each case) the corresponding iodoalkane or benzyl bromide/chloride (1.2 eq) (indicated in each case) was added dropwise. The mixture was stirred under argon at 40 °C for a period of time (indicated in each case) until completion, which was monitored by TLC. Work‐up and purification methods are also indicated in each case.

*Standard Procedure B* for the Sonogashira coupling with TMSA and subsequent alkyne‐TMS group deprotection. A dry THF/Et_3_N (4 : 1) solvent mixture was subjected to deoxygenation by three freeze‐pump‐thaw cycles with argon. Then, this solvent was added to the system containing the corresponding halogenated base (1 eq), CuI (0.01 eq) and Pd(PPh_3_)_2_Cl_2_ (0.02 eq). The mixture was stirred at room temperature for a few minutes. Then, trimethylsilylacetylene (TMSA; 2 eq) was added dropwise. The reaction was stirred under argon at a given temperature for a period of time (indicated in each case) until completion, which was monitored by TLC. Then, the mixture was filtrated over celite and the solvent evaporated under vacuum. The resulting crude was placed in a round‐bottom flask equipped with a magnetic stirrer, followed by addition of THF and the mixture was stirred at room temperature until the solid was dissolved. Then, hydrated tetrabutylammonium fluoride (TBAF ⋅ 3H_2_O; 1 eq) was slowly added at 0 °C. The mixture was allowed to reach room temperature and it was stirred until its completion, which was monitored by TLC (approximately 1 hour in all cases). The solvent was evaporated at reduced pressure and the product was purified by column chromatography (eluent indicated in each case). The resulting solid was finally washed with cold acetonitrile.

*Standard Procedure C* for the Sonogashira coupling with ethynyl‐nucleobases. A dry THF or THF/NEt_3_ solvent mixture (indicated in each case) was subjected to deoxygenation by three freeze‐pump‐thaw cycles with argon. Then, this solvent was added over the system containing the corresponding ethynyl‐substituted base (quantity indicated in each case), iodoarene derivative (quantity indicated in each case), Cul (0.01 eq) and PdCl_2_(PPh_3_)_2_ or Pd(Ph_3_)_4_ (0.02 eq). The mixture was stirred under argon at 40 °C (unless indicated otherwise) until completion, which was monitored by TLC. The purification methods are explained in each case.

**Starting materials**. Chemicals were purchased from commercial suppliers and used without further purification. Solid hygroscopic reagents were dried in a vacuum oven before use. Reaction solvents were thoroughly dried before use using standard methods. The synthesis and characterization of compounds **W**,^[19]^**W0**,^[10]^**W2**,^[11]^**C2.1**,^[9]^**C**,^[9]^**B**,^[12]^**B1**,^[13]^**G**^[9]^ and **GC1** ^[6a]^ have already been described in the literature.

### Synthetic procedures and characterization data of the nucleobase substituents (W2*, Scheme S1)

W2.1*: To a solution of the acid **W**^[19]^ (3.0 g, 4.5 mmol) in dry CH_2_Cl_2_ (50 mL) at 0 °C, EDC (1.7 g, 9.0 mmol) and HOBt (1.2 g, 9.0 mmol) were added, and then the solution was stirred for ten minutes. In another flask, a solution of the aminoacid (1.1 g, 6.7 mmol) and Et_3_N (1.2 mL, 9.0 mmol) in CH_2_Cl_2_ (40 mL) was stirred for 15 minutes, and then added to the solution of the acid. Once the addition was completed the ice bath was removed, the resulting solution was stirred at room temperature overnight. Once the reaction was completed, the solution was diluted with CH_2_Cl_2_ and then washed with HCl (0.1 N), NaHCO_3_ (sat) and NaCl (sat). The phases were separated and the organic layer was dried with Na_2_SO_4_. Finally, the solvent was evaporated under reduced pressure, and the resulting product was purified by column chromatography using AcOEt/Cyclohexane (1 : 6) as eluent. The desired product was obtained as a white solid (3.2 g, 90 %). ^1^H‐NMR (300 MHz, CDCl_3_) *δ*=6.99 (s, 2H, *H* ^*1*^), 6.52 (d, *J*=8.7 Hz, 1H, N*H*), 4.75 (dd, *J*=8.6, 5.0 Hz, 1H, C*H* ^*2*^), 4.00 (q, *J*=6.8 Hz, 6H, OC*H*_2_), 3.77 (s, 3H, OC*H*_3_), 2.26 (td, *J*=6.9, 5.0 Hz, 1H, *H* ^*3*^), 1.77 (ddd, *J*=21.3, 8.5, 6.3 Hz, 6H, OCH_2_C*H*_2_), 1.60–1.27 (m, 54H, OCH_2_CH_2_(C*H*_2_)_9_CH_3_), 0.99 (t, *J*=6.8 Hz, 6H, C*H*_3_^4^), 0.93–0.81 (m, 9H, O(CH_2_)_11_C*H*_3_) ppm. ^13^C‐NMR (75 MHz, CDCl_3_) *δ*=172.7, 167.0, 153.0, 141.5, 128.9, 105.9, 73.4, 69.3, 57.5, 52.0, 31.9, 31.5, 30.2, 29.7, 29.61, 29.57, 29.55, 29.50, 29.31, 29.28, 26.0, 22.6, 18.9, 18.0, 14.0 ppm. MS (ESI^+^): Calculated for C_49_H_89_NO_6_: 788.23; found: 789.69 [M+H]^+^. [α]D_20_=+11.78 (c=1, CHCl_3_).

W2*: To a suspension of the ester W2.1* (788.0 mg, 1.0 mmol) in MeCN (40 mL) at room temperature, NaOH 2N (3.2 mL, 6.5 mmol) was added, and the solution was then heated at 40 °C overnight. Once the reaction was completed, the solvent was removed under reduced pressure. The crude product was dissolved in CH_2_Cl_2_ and the solution was acidified until pH 3 was reached. The solution was then washed with NaCl and dried with Na_2_SO_4_. The evaporation of the solvent led to the final product (735.3 mg, 95 %). ^1^H‐NMR (300 MHz, CDCl_3_): *δ*=9.57 (s broad, 1H, O*H*), 6.99 (s, 2H, *H* ^*1*^), 6.62 (dd, *J*=8.6, 2.4 Hz, 1H, N*H*), 4.75 (dd, *J*=8.4, 4.8 Hz, 1H, *H* ^*2*^), 3.99 (td, *J*=6.5, 4.9 Hz, 6H, OC*H*_2_), 2.42–2.26 (m, 1H, *H* ^*3*^), 1.96–1.67 (m, 6H, OCH_2_C*H*_2_), 1.46 (dd, *J*=10.2, 5.3 Hz, 6H, OCH_2_CH_2_C*H*_2_), 1.40–1.21 (m, 48H, OCH_2_CH_2_CH_2_ (C*H*_2_)_8_CH_3_), 1.03 (t, *J*=6.9 Hz, 6H, C*H*_3_^4^), 0.97–0.79 (m, 9H, O(CH_2_)_11_C*H*_3_) ppm. ^13^C‐NMR (75 MHz, CDCl_3_) *δ*=175.4, 167.6, 152.9, 141.5, 128.3, 105.8, 73.3, 69.2, 57.4, 31.6, 31.0, 30.0, 29.42, 29.40, 29.38, 29.36, 29.34, 29.3, 29.12, 29.08, 29.06, 25.81, 25.78, 22.4, 18.8, 17.7, 13.8 ppm. MS (ESI^+^): Calculated for C_48_H_89_NO_6_: 775.67 [M+H]^+^ ; found: 775.67 [M+H]^+^. [α]D_20_=+14.89 (c=1, CHCl_3_).

### Synthetic procedures and characterization data for the new cytosine derivatives (C0, C2 and C2*, Schemes 1 and S2)

**C0.1**. Following *Standard Procedure A*, to a solution of **C**^[9]^ (1.0 g, 4.2 mmol) in dry DMF (100 mL), a 1.0 M solution of Bu_4_NOH in MeOH was added (4.2 mL, 4.2 mmol) and the mixture was stirred for 30 minutes at 50 °C. Then, 1‐(bromomethyl)‐3,4,5‐tris(dodecyloxy)benzene (**W0**)^[10]^ (3.4 g, 4.6 mmol) was dissolved in dry DMF (50 mL) and added *via cannula* to the solution. This solution was stirred for 12 h. After completion, the solvent was evaporated under reduced pressure. The residue was purified by column chromatography eluted with CHCl_3_:MeOH (20 : 1) to afford **C0.1** as a white solid (2.8 g, 76 %). ^1^H‐NMR (300 MHz, CDCl_3_): *δ*=9.10 (s broad, 1H, C^4^NH‐*H*), 7.48 (s, 1H, *H* ^*6*^), 6.46 (s, 2H, s, *H* ^*2*^), 5.65 (s, 1H, C^4^NH‐*H*), 4.82 (s, 2H, N^1^C*H*_2_), 3.92 (t, *J*=6.3 Hz, 6H, OC*H*_2_), 1.75 (m, 6H, OCH_2_C*H*_2_), 1.24 (m, 54H, OCH_2_CH_2_(C*H*_2_)_9_CH_3_), 0.86 (m, 9H, O(CH_2_)_11_C*H*_3_) ppm. ^13^C‐NMR (75 MHz, CDCl_3_) *δ*=165.0, 155.1, 153.6, 148.3, 138.4, 130.6, 107.1, 90.2, 84.0, 77.3, 75.0, 73.4, 69.3, 52.7, 32.0, 30.4, 29.8, 29.7, 29.5, 26.17, 22.74, 14.2 ppm. HRMS (ESI^+^): Calculated for C_47_H_83_IN_3_O_4_: 880.5422 [M+H]^+^. Found: 880.5454 [M+H]^+^. m. p. 115.4–117.7 °C.

**C0**. Following *Standard Procedure B*, to a solution of **C0.1** (500.0 mg, 0.6 mmol), Pd(Ph_3_)_2_)Cl_2_ (7.7 mg, 0.01 mmol), and CuI (1.1 mg, 0.01 mmol) in THF/Et_3_N (5 mL), TMSA (1.4 mL, 2.3 mmol) was added and the mixture stirred at 40 °C overnight. Once the reaction was completed, the solvent was evaporated, the resulting crude was suspended in THF (10 mL) and TBAF ⋅ 3H_2_O (625.0 mg, 0.6 mmol) was added. After completion the solvent was evaporated and the resulting residue was purified by column chromatography eluted with CHCl_3_:MeOH (50 : 1), affording **C0** as a pale solid (340.0 mg, 77 %). ^1^H‐NMR (300 MHz, CDCl_3_): *δ*=7.47 (s, 1H, *H* ^6C^), 6.87 (s broad, 1H, C^4^NH‐*H*), 6.49 (s, 2H, *H* ^*2*^), 5.67 (s broad, 1H, C^4^NH‐*H*), 4.87 (s, 2H, N^1^C*H*_2_), 3.93 (td, *J*=6.5, 2.2 Hz, 6H, OC*H*_2_), 3.32 (s, 1H, C≡C*H*), 1.89–1.52 (m, 6H, OCH_2_C*H*_2_), 1.28 (m, 54H, OCH_2_CH_2_(C*H*_2_)_9_CH_3_), 1.03–0.67 (m, 9H, O(CH_2_)_11_C*H*_3_) ppm. ^13^C‐NMR (75 MHz, CDCl_3_): *δ*=165.0, 155.1, 153.6, 148.3, 138.4, 130.6, 107.1, 90.2, 84.0, 77.3, 75.0, 73.4, 69.3, 52.7, 32.0, 30.4, 29.8, 29.7, 29.5, 26.2, 22.7, 14.2 ppm. HRMS (ESI^+^): Calculated for C_49_H_84_N_3_O_4_: 778.6456 [M+H]^+^. Found: 778.6464 [M+H]^+^. m. p. 94.2–95.9 °C.

**C2**. To a suspension of **C2.1**^[9]^ (270.0 mg, 1.0 mmol) in CH_2_Cl_2_ (26 mL), TFA (4.0 mL, 51.4 mmol) was added. The resulting solution was stirred at room temperature for 3 h, then TFA was removed by coevaporation with CH_2_Cl_2_ under reduced pressure. The resulting crude was re‐dissolved in dry DMF and stirred at room temperature for 20 minutes, then cooled to 0 °C. Meanwhile, to a solution of the acid **W2** ^[11]^ (710.0 mg, 1.0 mmol) in CH_2_Cl_2_ at 0 °C, EDC (267.2 mg, 1.9 mmol), HOBt (331.2 mg, 2.5 mmol) and DIPEA (0.3 mL, 1.9 mmol) were added. The solution was stirred at room temperature for one hour, the solvent was removed under reduced pressure and the crude re‐dissolved with DMF and a few drops of a CHCl_3_:MeOH (10 : 1) mixture. This solution was added to the solution at 0 °C of the deprotected amine, and then stirred at room temperature overnight. Once the reaction was completed, the solution was washed with HCl (0.1 M), NaHCO_3_ (sat) and NaCl (sat). The phases were separated and the organic layer was dried with MgSO_4_ and the solvent evaporated under reduced pressure. The crude product was purified by column chromatography using a 10 : 1 CHCl_3_:MeOH mixture as eluent, affording a white solid (89.1 mg, 50 %). ^1^H‐NMR (300 MHz, CDCl_3_): *δ*=8.71 (s broad, 1H, N*H* ^*5*^), 7.81 (s broad,1H, N*H* ^*3*^), 7.46 (s, 1H, *H* ^*6C*^), 7.08 (s, 2H, *H* ^*6*^), 4.21 (s broad, 2H, C*H*_2_^4^), 4.19–3.87 (m, 8H, OCH_2_, C*H*_2_^2^), 3.59 (s broad, 2H, C*H*_2_^1^), 3.25 (s, 1H, C≡C*H*), 1.79–1.67 (m, 6H, OCH_2_C*H*_2_), 1.45–1.13 (m, 54H, OCH_2_CH_2_(C*H*_2_)_9_CH_3_), 0.88–0.84 (m, 9H, O(CH_2_)_11_C*H*_3_) ppm. ^13^C‐NMR (75 MHz, CDCl_3_): *δ*=170.6, 168.1, 165.0, 155.6, 153.2, 150.5, 141.7, 128.7, 106.3, 90.0, 83.9, 75.3, 73.7, 69.6, 50.2, 43.8, 38.1, 32.1, 30.5, 29.91, 29.87, 29.85, 29.82, 29.75, 29.6, 29.54, 29.52, 26.29, 26.25, 22.9, 14.7. MS (ESI^+^): Calculated for C_53_H_90_N_5_O_6_ [M+H]^+^: 892.68; found: 892.66 [M+H].

C2*: To a suspension of **C2.1** (250.0 mg, 0.9 mmol) in CH_2_Cl_2_ (15 mL), TFA was added (3.0 mL, 0.04 mmol), the resulting solution was stirred at room temperature for 3.5 h. Then, TFA was removed by coevaporation with CH_2_Cl_2_. The resulting solid was re‐dissolved in CH_2_Cl_2_ (25 mL), then Et_3_N (276.0 μL, 2.0 mmol) was added and the solution was stirred for 15 minutes. In another flask to a solution of the acid W2* (465.0 mg, 0.6 mmol) in dry CH_2_Cl_2_ (25 mL) at 0 °C, HOBt (162.0 mg, 1.2 mmol) and EDC (230.0 mg, 1.2 mmol) were added. The mixture was stirred for 15 minutes, then, the solution of the deprotected amine was added. The mixture was stirred at room temperature overnight. Once the reaction was completed, the solution was diluted with CH_2_Cl_2_ and then washed with HCl (0.1 N), NaHCO_3_ (sat) and NaCl (sat). The phases were separated, and the organic layer was dried with Na_2_SO_4_ and then the solvent was evaporated. The crude product was purified by column chromatography, eluted with CHCl_3_: MeCN (30 : 1) obtained C2* as a white solid (404.0 mg, 48 %). ^1^H‐NMR (300 MHz, CDCl_3_) *δ*=9.01 (m, 1H, N^3^ *H*), 7.87 (s broad, 1H, NH‐*H*), 7.27 (s, 1H, C*H* ^*6*^), 7.05 (s, 2H, *H* ^*6*^), 6.94 (d, *J*=9.3 Hz, 1H, N^5^ *H*), 5.56 (broad, 1H, NH‐*H*), 5.09 (dd, *J*=6.9, 9.3 Hz, 1H, *H* ^*4*^), 4.17–3.92 (m, 2H, C*H*_2_^2^), 3.60–3.58 (m, 2H, N^1^C*H*_2_) 4.02 (m, 6H, OC*H*_2_), 2.96 (s, 1H, C≡C*H*), 2.15 (m, 1H, *H* ^*7*^), 1.88–1.64 (m, 6H, OCH_2_C*H*_2_), 1.54–1.19 (m, 54H, OCH_2_CH_2_(C*H*_2_)_9_CH_3_), 1.04 (d, *J*=8.2 Hz, 3H, C*H*_3_^8^), 1.02 (d, *J*=8.2 Hz, 3H, C*H*_3_^9^), 0.89 (m, 9H, O (C*H*_2_)_11_CH_3_) ppm. ^13^C‐NMR (75 MHz, CDCl_3_) *δ*=172.8, 167.5, 164.9, 155.5, 153. 3, 150.5, 141.8, 129.3, 106.5, 89.8, 83.4, 77.3, 75.4, 73.8, 69.8, 58.7, 49.8, 37.20, 32.26, 32.08, 32.07, 30.5, 29.89, 29.85, 29.80, 29.74, 29.61, 29.59, 29.53, 29.51, 26.28, 26.25, 22.8, 19.5, 18.65, 14.24 ppm. MS (ESI^+^): Calculated for C_56_H_95_N_5_O_6_ [M+H]^+^: 934.40; found: 935.75 [M+H]^+^. [α]D_20_=−1.21 (c=1, CHCl_3_).

### Synthetic procedures and characterization data for the new G−C final monomers (GC0, GC2 and GC2*, Schemes 1 and S3)

**BC0**: Following *Standard Procedure C*, **B**^[12]^ (1.0 g, 1.56 mmol), **C0** (403.0 mg, 0.51 mmol), Pd(Ph_3_)_2_Cl_2_ (6.8 mg, 0.01 mmol) and CuI (0.9 mg, 0.005 mmol) were dissolved in a THF:Et_3_N mixture (15 mL). the resulting mixture was stirred at 40 °C overnight. Once the reaction was completed, the solvent was removed under reduced pressure. The resulting product was purified by column chromatography, using as eluent a CHCl_3_:MeOH 50 : 1 mixture. The product was obtained as a colorless oil (345 mg, 55 %).^1^H‐NMR (300 MHz, CDCl_3_): *δ* =7.41 (s, 1H, *H* ^*6C*^), 7.28 (s, 1H, 1*H* ^*b*^), 6.85 (s, 1H, *H* ^*a*^), 6.78 (s broad, 1H, NH‐*H*), 6.49 (s, 2H, *H* ^*2*^), 6.16 (bs, 1H, NH‐*H*), 4.89 (s, 2H, C*H*_2_^2^), 4.04–3.89 (m, 10H, OC*H*_2_(CH_2_)_10_CH_3_, C*H*_2_^*c*^), 1.95–1.69 (m, 10H, OCH_2_C*H*_2_(CH_2_)_9_CH_3_, C*H*_2_^*d*^), 1.67–1.52 (m, 2H, *H*_j_), 1.46–1.08 (m, 70H, OCH_2_CH_2_(C*H*_2_)_9_CH_3_, C*H*_2_^*f,g,h*^, *H* ^*e,j*^), 1.01–0.76 (m, 27H, O(CH_2_)_11_C*H*_3,_ C*H*_3_^*k,I,l*^). HRMS (ESI^+^): Calculated for C_75_H_127_IN_3_O_6_: 1292.8764 [M+H]^+^. Found: 1292.8772 [M+H]^+^.

**BC2**: Following *Standard Procedure C*, **C2** (100.0 mg, 0.11 mmol), **B** ^[12]^ (115.3 mg, 0.34 mmol), Pd(Ph_3_)_2_Cl_2_ (1.57 mg, 0.002 mmol) and CuI (0.2 mg, 0.001 mmol) were dissolved in a THF:Et_3_N mixture. The solution was stirred at 40 °C overnight. The solvent was then removed under reduced pressure, and the resulting product was purified by column chromatography, using as eluent a CHCl_3_:MeOH 100 : 1 solvent mixture. The product was obtained as a colorless oil (74.1 mg, 47 %). ^1^H‐NMR (300 MHz, CDCl_3_): *δ*=8.32 (s broad, 1H, N*H* ^*5*^), 7.44 (s, 1H, *H* ^*6C*^), 7.09 (s, 1H, *H* ^*b*^), 7.10 (s, 1H, *H* ^*a*^), 7.06 (s, 2H, *H* ^*6*^), 7.01 (s broad, 1H, NH‐*H*), 6.75 (s, 1H, N*H* ^*3*^), 6.25 (s broad, 1H, NH‐*H*), 4.22 (qd, *J*=16.3, 5.7 Hz, 2H, C*H*_2_^4^), 4.02–3.89 (m, 10H, OC*H*_2_(CH_2_)_10_CH_3_, C*H*_2_^*c*^, C*H*_2_^*2*^), 3.76–3.60 (m, 4H, C*H*_2_^*d*^), 1.80–1.67 (m, 6H, OCH_2_C*H*_2_(CH_2_)_9_CH_3_), 1.65–1.51 (m, 8H, *H* ^*e*^, C*H*_2_^*f*^, C*H*_2_^*1*^), 1.51–0.99 (m, 66H, OCH_2_CH_2_(C*H*_2_)_9_CH_3_, C*H*_2_^*g,h*^, *H* ^*j*^), 0.86 (m, 27H, O(CH_2_)_11_C*H*_3_, C*H*_3_^*l,I,k*^). HRMS (MALDI+): Calculated for C_79_H_132_N_5_O_8_I: 1406.9193 [M+H]^+^. Found: 1406.9187 [M+H]^+^.

B1C2*: Following *Standard Procedure C* for a Sonogashira coupling, C2* (200.0 mg, 0.214 mmol), **B1** ^[13]^ (627.2 mg, 1.07 mmol), Pd(Ph_3_)_2_Cl_2_ (3.0 mg, 0.004 mmol) and CuI (0.4 mg, 0.0002 mmol) were dissolven in a THF:Et_3_N mixture (10 mL). The resulting solution was stirred at r.t. overnight, and then heated at 50 °C for 1 h. Once the reaction was completed, the solvent was evaporated under reduced pressure. The residue was then purified by column chromatography, using as eluent a CHCl_3_:MeOH (100 : 1) mixture. The product was obtained as a white solid (140.0 mg, 47 %). ^1^H‐NMR (300 MHz, CDCl_3_) *δ*=9.01 (m, 1H, N^3^ *H*), 7.87 (s broad, 1H, NH‐*H*), 7.27 (s, 1H, C*H* ^*6*^), 7.20 (s, 1H, *H* ^*b*^), 7.05 (s, 2H, *H* ^*6*^), 7.03 (s, 2H, *H* ^*a*^), 6.94 (d, *J*=9.3 Hz, 1H, N^5^ *H*), 5.56 (broad, 1H, NH‐*H*), 5.09 (dd, *J*=6.9, 9.3 Hz, 1H, *H* ^*4*^), 4.17–3.92 (m, 2H, C*H*_2_^*2*^), 3.60–3.58 (m, 2H, N^1^C*H*_2_) 4.02 (m, 10H, OC*H*_2_), 2.15 (m, 1H, *H* ^*7*^), 1.88–1.64 (m, 10H, OCH_2_C*H*_2_), 1.54–1.19 (m, 74H, OCH_2_CH_2_(C*H*_2_)_9_CH_3_, OCH_2_CH_2_(C*H*_2_)_5_CH_3_), 1.04 (d, *J*=8.2 Hz, 3H, C*H*_3_^*8*^), 1.02 (d, *J*=8.2 Hz, 3H, C*H*_3_^*9*^), 0.89 (m, 15H, O(CH_2_)_11_C*H*_3,_ O(CH_2_)_7_C*H*_3_) ppm.

**GC0**: Following *Standard Procedure C* for a Sonogashira coupling, **BC0** (100.0 mg, 0.082 mmol), **G** ^[9]^ (80.0 mg, 0.097 mmol), Pd(Ph_3_)_4_ (2.226 mg, 0.002 mmol) and CuI (0.2 mg, 0.001 mmol) were dissolved in a THF:Et_3_N mixture (3 mL). The mixture was stirred at 40 °C for 12 h. Once the reaction was completed, the solvent was evaporated under reduced pressure. The crude product was purified by column chromatography, using as eluent a CHCl_3_:MeOH 100 : 1 mixture. The product was obtained as a yellow solid (48.9 mg, 30 %). ^1^H‐NMR (300 MHz, CDCl_3_): *δ*=7.64 (s, 1H, NH‐*H*), 7.41 (s, 1H, *H* ^*6C*^), 7.13 (s, 1H, *H* ^*b*^), 7.01 (s, 1H, *H* ^*a*^), 6.79 (s, 2H, *H* ^*2’*^), 6.53 (s, 2H, *H* ^*2*^), 5.38 (s, 3H, C*H*_2_^*1’*^), 4.94 (s, 2H, C*H*_2_^*1*^), 4.21–3.82 (m, 20H, OC*H*_2_(CH_2_)_10_CH_3_, C*H*_2_^*c,d*^), 1.94–1.01 (m, 136H, OCH_2_(C*H*_2_)_10_CH_3_), C*H*_2_^,*f,g,h*^ *, H* ^*e*^, *H^j^)*, 1.01–0.75 (m, 36H, O(CH_2_)_11_C*H*_3_, C*H*_3_^*i,k,l*^). HRMS (MALDI+): Calculated for C_127_H_211_N_9_O_11_Na: 2062.6153 [M+Na]^+^. Found: 2062.6219 [M+Na]^+^.

**GC2**: Following *Standard Procedure C* for a Sonogashira coupling **BC2** (100.0 mg, 0.071 mmol), **G** ^[9]^ (70.0 mg, 0.085 mmol), Pd(Ph_3_)_4_ (1.15 mg, 0.001 mmol) and CuI (0.1 mg, 0.0007 mmol) were dissolved in a THF:Et_3_N mixture (3 mL) and stirred at 40°C overnight. Once the reaction was completed, the solvent was removed under reduced pressure. The residue was then purified by column chromatography, using as eluent a CHCl_3_:MeOH 100 : 1 mixture. The product was obtained as a yellow solid (100.5 mg, 67 %). ^1^H‐NMR (300 MHz, CDCl_3_): *δ*=7.98 (s broad, 1H N*H* ^*5*^), 7.75 (s broad, 1H, N*H* ^*3*^), 7.51 (s, 1H, *H* ^*6C*^), 7.13 (s, 1H, *H* ^*b*^), 7.03 (s, 1H, *H* ^*a*^), 6.99 (s, 2H, *H* ^*6*^), 6.80 (s, 2H, *H* ^*2’*^), 5.39 (s, 2H, C*H*_2_^*1’*^), 4.34–3.94 (m, 20H, OC*H*_2_(C*H*_2_)_10_CH_3_, C*H*_2_^*c,d*^), 3.89–3.70 (m, 6H, C*H*_2_^*4,2,1*^), 1.74–1.50 (m, 12H, C*H*_2_^,*f,g,h*^), 1.49–0.96 (m, 112 H, OCH_2_CH_2_(C*H*_2_)_9_CH_3_, *H* ^*e*^, *H* ^*j*^), 0.87 (m, 36H, O(CH_2_)_11_C*H*_3_, C*H*_3_^*i,k,l*^) ppm. HRMS (ESI^+^): Calculated for C_129_H_215_N_10_O_12_: 2097.6548 [M+H]^+^. Found: 2097.6566 [M+H]^+^.

GC2*: Following *Standard Procedure C* for a Sonogashira coupling, B1C2* (101.7 mg, 0.071 mmol, **G** ^[9]^ (72 mg, 0.087 mmol), Pd(Ph_3_)_4_ (1.15 mg, 0.001 mmol) and CuI (0.13 mg, 0.0007 mmol) in THF. Then a few drops of ^i^Pr_2_NH were added, and the solution was stirred at r.t. overnight. Once the reaction was completed, the mixture was filtered over a celite plug and washed with CHCl_3_:MeOH 10 : 1. The solvent was then evaporated under reduced pressure and the residue was purified by column chromatography, using as eluent CHCl_3_:MeOH 50 : 1. The product was obtained as a yellow solid (16 mg, 10 %). ^1^H‐NMR (300 MHz, CDCl_3_, TFA 1 %) *δ*=7.96 (s, 1H, *H* ^*6C*^), 7.54 (s, 1H, *H* ^*b*^), 7.12 (s, 1H, *H* ^*a*^), 7.04 (s, 2H, *H* ^*6*^), 7.00 (s broad, 1H, N*H* ^*3*^), 6.80 (s, 2H, *H* ^*2’*^), 5.40 (s, 2H, C*H*_2_^*1’*^), 4.23–3.93 (m, 17H, OC*H*_2_(CH_2_)_10_CH_3_, *H* ^*4*^, OC*H*_2_(CH_2_)_6_CH_3_), 3.90–3.71 (m, 4H, C*H*_2_^*2*^, C*H*_2_^*1*^), 1.85–1.72 (m, 16H, OCH_2_C*H*_2_(CH_2_)_9_CH_3_, OCH_2_C*H*_2_(CH_2_)_5_CH_3_), 1.51–1.00 (m, 129H, OCH_2_CH_2_(C*H*_2_)_9_CH_3_, OCH_2_CH_2_(C*H*_2_)_5_CH_3_, *H* ^*7*^), 1.11–0.73 (m, 30H, O(CH_2_)_11_C*H*_3_, O(CH_2_)_7_C*H_3_*, C*H*_3_^*8*^, C*H*_3_^*9*^) ppm. HRMS (ESI^+^): Calculated for C_128_H_212_N_10_O_12_: 2083.6391 [M+H]^+^. Found: 2083.6391 [M+H]^+^.

## Conflict of interest

The authors declare no conflict of interest.

## Supporting information

As a service to our authors and readers, this journal provides supporting information supplied by the authors. Such materials are peer reviewed and may be re‐organized for online delivery, but are not copy‐edited or typeset. Technical support issues arising from supporting information (other than missing files) should be addressed to the authors.

Supporting Information
