# Supplementary material for: The Role of Peripheral Amide Groups as Hydrogen‐Bonding Directors in the Tubular Self‐Assembly of Dinucleobase Monomers
Source: Chempluschem. 2021 Jun 29;86(8):1087–96. doi: 10.1002/cplu.202100255 (PMC8457134; doi:10.1002/cplu.202100255)
Supplement: Supplementary file 1 — Supporting Information [file CPLU-86-1087-s001.pdf]

ChemPlusChem

Supporting Information

The Role of Peripheral Amide Groups as Hydrogen-Bonding Directors in the Tubular Self-Assembly of Dinucleobase Monomers

Violeta Vázquez-González, María J. Mayoral, Fátima Aparicio, Paula Martínez-Arjona, and David González-Rodríguez*

TABLE OF CONTENTS

S0. General Methods and Synthetic Procedures 2
S1. UV-vis and emission spectra in diverse solvents 15
S2. Monomer-Cyclic Tetramer Equilibria Studied in THF 16
  S2.1. Temperature-dependent Experiments 16
  S2.2. Concentration -dependent Experiments 18
  S2.3. Solvent -dependent Experiments 19
  S2.4. Quantitative Analysis of the Cyclotetramerization Process in THF 20
S3. Calculation of Reference G:C Association Constants 23
S4. Cyclic Tetramer-Polymer Equilibria Studied in Apolar Aliphatic Solvents 25
S5. Characterization of the Self-assembled Nanotubes by TEM 31
S6. References 33

S0. General Methods and Synthetic Procedures

General Methods. Mass Spectrometry (MS) and **High Resolution-Mass Spectrometry (HRMS)** MALDI-TOF spectra were obtained from a BRUKER ULTRAFLEX III instrument equipped with a nitrogen laser operating at 337 nm. **NMR** spectra were recorded with a *BRUKER AVANCE-II* 300 MHz or a *BRUKER DRX* 500 MHz instrument. The temperature was actively controlled at 298 K. Chemical shifts are measured in ppm using the signals of the deuterated solvent as the internal standard [$CDCl_3$ calibrated at 7.26 ppm (^1H) and 75.0 ppm (^{13}C), DMSO-d_6 calibrated at 2.50 ppm (^1H) and 39.5 ppm (^{13}C) and THF-d_8 calibrated at 3.58 (^1H) and 39.5 ppm (^{13}C)]. Due to solubility problems the ^{13}C-NMR data for some final monomers and their derivatives could not be performed. **Column chromatography** was carried out on silica gel *Merck-60* (230-400 mesh, 60 Å), and TLC on aluminium sheets precoated with silica gel 60 F254 (Merck). **FT-IR** spectra were recorded with a PerkinElmer spectrometer UATR two. **UV-Visible** experiments were conducted using a *JASCO V-660* apparatus. **Emission spectra** were recorded in a *JASCO FP-8600* equipment using excitation and emission bandwidths of 5 nm in both cases, and a 50 ms response. **CD spectra** were recorded with a *JASCO* J-815 equipment. The slit width was set at 1000 µm and a DIT of 2 s was used. In all these three instruments the temperature was controlled using a *JASCO* Peltier thermostatted cell holder with a range of 263–383 K, adjustable temperature slope, and accuracy of ± 0.1 K. **Transmission electron microscopy** (**TEM**) images were obtained with a *JEOL-JEM 1010* instrument operating at 100 kV for the stained samples and a *JEM 1400 K PLUS* instrument operating at 100 kV for non-stained samples.

Standard procedures

- *Standard Procedure A* for the nucleobase alkylation reaction. To a suspension of the nucleobase starting material (1 eq) and a base (1.2 eq) (indicated in each case) in dry DMF (volume indicated in each case) the corresponding iodoalkane or benzyl bromide/chloride (1.2 eq) (indicated in each case) was added dropwise. The mixture was stirred under argon at 40 C for a period of time (indicated in each case) until completion, which was monitored by TLC. Work-up and purification methods are also indicated in each case.
- *Standard Procedure B* for the Sonogashira coupling with TMSA and subsequent alkyne-TMS group deprotection. A dry THF/Et_3N (4:1) solvent mixture was subjected to deoxygenation by three freeze-pump-thaw cycles with argon. Then, this solvent was added to the system containing the corresponding halogenated base (1 eq), CuI (0.01 eq) and $Pd(PPh_3)_2Cl_2$ (0.02 eq). The mixture was stirred at room temperature for a few minutes. Then, trimethylsilylacetylene (TMSA; 2 eq) was added dropwise. The reaction was stirred under argon at a given temperature for a period of time (indicated in each case) until completion, which was monitored by TLC. Then, the mixture was filtrated over celite and the solvent evaporated under vacuum. The resulting crude was placed in a round-bottom flask equipped with a magnetic stirrer, followed by addition of THF and the mixture was stirred at room temperature until the solid was dissolved. Then, hydrated tetrabutylammonium fluoride (TBAF·$3H_2O$; 1 eq) was slowly added at 0 ºC. The mixture was allowed to reach room temperature and it was stirred until its completion, which was monitored by TLC (approximately 1 hour in all cases). The solvent was evaporated at reduced

pressure and the product was purified by column chromatography (eluent indicated in each case). The resulting solid was finally washed with cold acetonitrile.

- *Standard Procedure C* for the Sonogashira coupling with ethynyl-nucleobases. A dry THF or THF/NEt_3 solvent mixture (indicated in each case) was subjected to deoxygenation by three freeze-pump-thaw cycles with argon. Then, this solvent was added over the system containing the corresponding ethynyl-substituted base (quantity indicated in each case), iodoarene derivative (quantity indicated in each case), CuI (0.01 eq) and $PdCl_2(PPh_3)_2$ or $Pd(Ph_3)_4$ (0.02 eq). The mixture was stirred under argon at 40 ºC (unless indicated otherwise) until completion, which was monitored by TLC. The purification methods are explained in each case.

Starting materials

Chemicals were purchased from commercial suppliers and used without further purification. Solid hygroscopic reagents were dried in a vacuum oven before use. Reaction solvents were thoroughly dried before use using standard methods. The synthesis and characterization of compounds **W**,[1] **W0**,[2] **W2**,[3], **C2.1**,[4] **C**,[4] **B,**[5] **B1,**[6] **G**[4] and **GC1**[7] have already been described in the literature.

Synthetic procedures and characterization data of the nucleobase substituents (W2*)

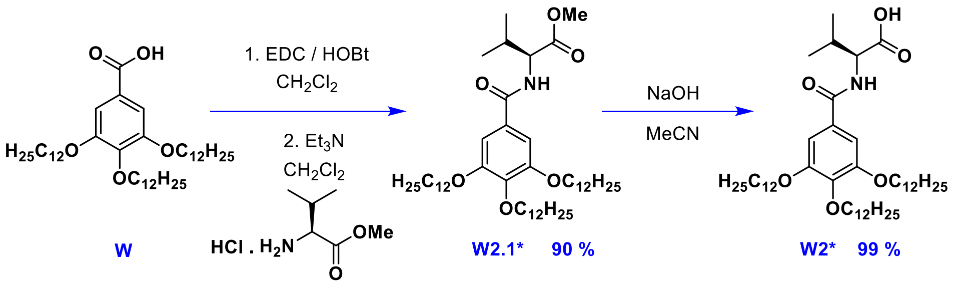

Scheme S1.

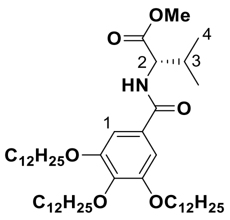

W2.1*. To a solution of the acid **W**[1] (3.0 g, 4.5 mmol) in dry CH_2Cl_2 (50 mL) at 0 ºC, EDC (1.7 g, 9.0 mmol) and HOBt (1.2 g, 9.0 mmol) were added, and then the solution was stirred for ten minutes. In another flask, a solution of the aminoacid (1.1 g, 6.7 mmol) and Et_3N (1.2 mL, 9.0 mmol) in CH_2Cl_2 (40 mL) was stirred for 15 minutes, and then added to the solution of the acid. Once the addition was completed the ice bath was removed, the resulting solution was stirred at room temperature overnight. Once the reaction was completed, the solution was diluted with CH_2Cl_2 and then washed with HCl (0.1 N), $NaHCO_3$ (sat) and NaCl (sat). The phases were separated and the organic layer was dried with Na_2SO_4. Finally, the solvent was evaporated under reduced pressure, and the resulting product was purified by column chromatography using AcOEt/Cyclohexane (1:6) as eluent. The desired product was obtained as a white solid (3.2 g, 90%). **^{1}H-NMR** (300 MHz, $CDCl_3$) δ =6.99 (s, 2H, H^1), 6.52 (d, J = 8.7 Hz, 1H, NH), 4.75 (dd, J = 8.6, 5.0 Hz, 1H, CH^2), 4.00 (q, J = 6.8 Hz, 6H, OCH_2), 3.77 (s, 3H, OCH_3), 2.26 (td, J = 6.9, 5.0 Hz, 1H, H^3), 1.77 (ddd, J = 21.3, 8.5, 6.3 Hz, 6H, OCH_2CH_2), 1.60–1.27 (m, 54H, $OCH_2CH_2(CH_2)_9CH_3$), 0.99 (t, J = 6.8 Hz, 6H, C$H_3{}^4$), 0.93–0.81 (m, 9H, $O(CH_2)_{11}CH_3$) ppm. **^{13}C-NMR** (75 MHz, $CDCl_3$) δ = 172.7, 167.0, 153.0, 141.5, 128.9, 105.9, 73.4, 69.3, 57.5, 52.0, 31.9, 31.5, 30.2, 29.7, 29.61, 29.57, 29.55, 29.50, 29.31, 29.28, 26.0, 22.6, 18.9, 18.0, 14.0 ppm. **MS** (ESI$^+$): Calculated for $C_{49}H_{89}NO_6$: 788.23; found: 789.69 $[M+H]^+$. **[α]$^D{}_{20}$** =+11.78 (c 1, $CHCl_3$).

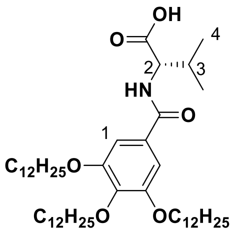

W2*. To a suspension of the ester **W2.1*** (788.0 mg, 1.0 mmol) in MeCN (40 mL) at room temperature, NaOH 2N (3.2 mL, 6.5 mmol) was added, and the solution was then heated at 40 ºC overnight. Once the reaction was completed, the solvent was removed under reduced pressure. The crude product was dissolved in CH_2Cl_2 and the solution was acidified until pH 3 was reached. The solution was then washed with NaCl and dried with Na_2SO_4. The evaporation of the solvent led to the final product (735.3 mg, 95%). **^{1}H-NMR** (300 MHz, $CDCl_3$): δ = 9.57 (s broad, 1H, OH), 6.99 (s, 2H, H^1), 6.62 (dd, J = 8.6, 2.4 Hz, 1H, NH), 4.75 (dd, J = 8.4, 4.8 Hz, 1H, H^2), 3.99 (td, J = 6.5, 4.9 Hz, 6H, OCH_2), 2.42–2.26 (m, 1H, H^3), 1.96–1.67 (m, 6H, OCH_2CH_2), 1.46 (dd, J = 10.2, 5.3 Hz, 6H, $OCH_2CH_2CH_2$), 1.40–1.21 (m, 48H, $OCH_2CH_2CH_2$ $(CH_2)_8CH_3$), 1.03 (t, J = 6.9 Hz, 6H, C$H_3{}^4$), 0.97–0.79 (m, 9H, $O(CH_2)_{11}CH_3$) ppm. **^{13}C-NMR** (75 MHz, $CDCl_3$) δ = 175.4, 167.6, 152.9, 141.5, 128.3, 105.8, 73.3, 69.2, 57.4, 31.6, 31.0, 30.0, 29.42, 29.40, 29.38, 29.36, 29.34, 29.3, 29.12, 29.08, 29.06, 25.81, 25.78, 22.4, 18.8, 17.7, 13.8 ppm. **MS** (ESI$^+$): Calculated for $C_{48}H_{89}NO_6$: 775.67 $[M+H]^+$; found: 775.67 $[M+H]^+$. **[α]$^D{}_{20}$** = +14.89 (c 1, $CHCl_3$).

Synthetic procedures and characterization data for the new cytosine derivatives (C0, C2 and C2*)

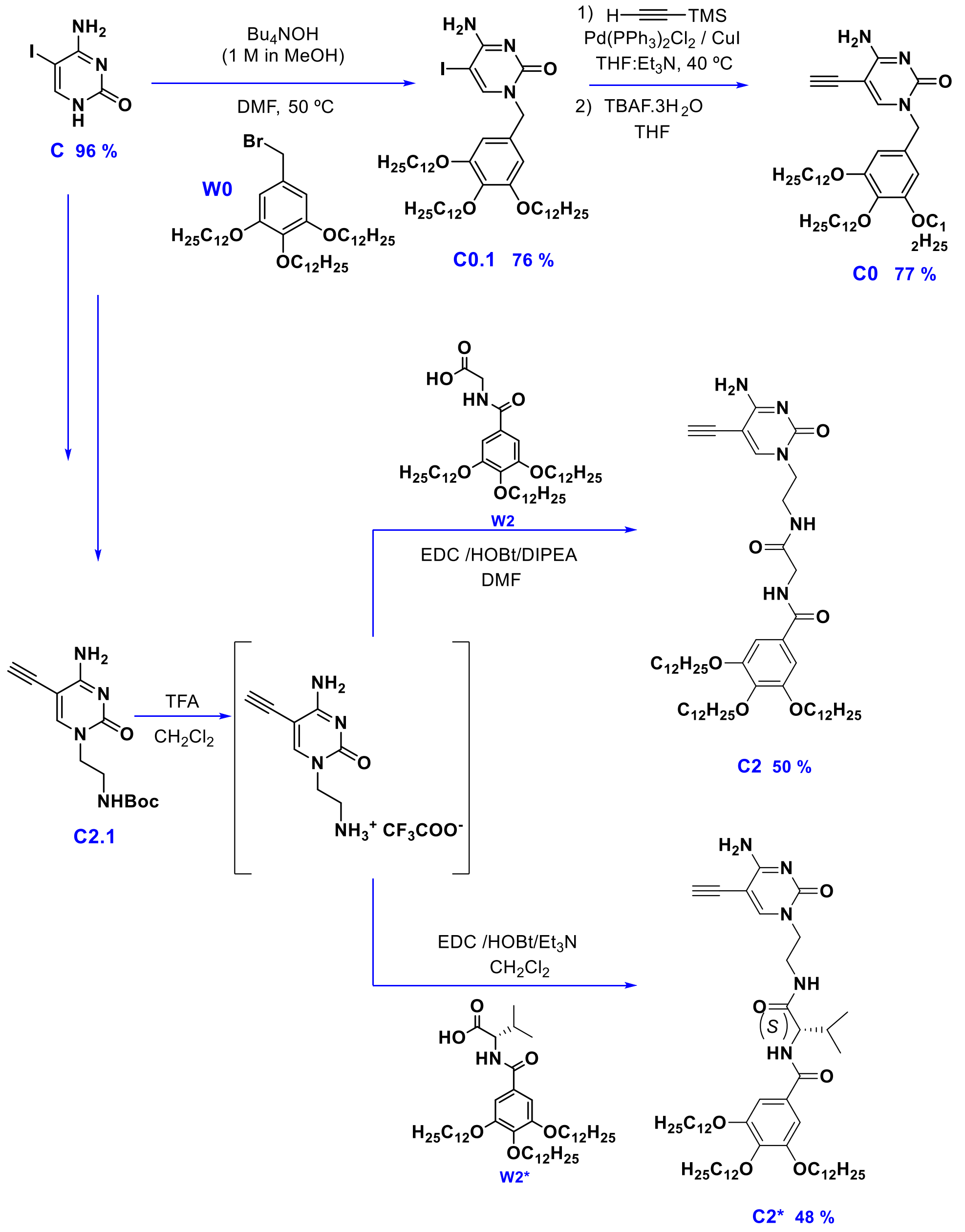

Scheme S2.

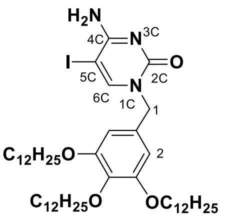

C0.1. Following *Standard Procedure A*, to a solution of **C**[4] (1.0 g, 4.2 mmol) in dry DMF (100 mL), a 1.0 M solution of Bu_4NOH in MeOH was added (4.2 mL, 4.2 mmol) and the mixture was stirred for 30 minutes at 50 ºC. Then, 1-(bromomethyl)-3,4,5-tris(dodecyloxy)benzene (**W0**)[2] (3.4 g, 4.6 mmol) was dissolved in dry DMF (50 mL) and added *via cannula* to the solution. This solution was stirred for 12 h. After completion, the solvent was evaporated under reduced pressure. The residue was purified by column chromatography eluted with $CHCl_3$:MeOH (20:1) to afford **C0.1** as a white solid (2.8 g, 76%). **^{1}H-NMR** (300 MHz, $CDCl_3$): δ = 9.10 (s broad, 1H, C^4NH-H), 7.48 (s, 1H, H^6), 6.46 (s, 2H, s, H^2), 5.65 (s, 1H, C^4NH-H),

4.82 (s, 2H, N^{1}CH_2), 3.92 (t, J = 6.3 Hz, 6H, OCH_2), 1.75 (m, 6H, OCH_2CH_2), 1.24 (m, 54H, $OCH_2CH_2(CH_2)_9CH_3$), 0.86 (m, 9H, $O(CH_2)_{11}CH_3$) ppm. **^{13}C-NMR** (75 MHz, $CDCl_3$) δ = 165.0, 155.1, 153.6, 148.3, 138.4, 130.6, 107.1, 90.2, 84.0, 77.3, 75.0, 73.4, 69.3, 52.7, 32.0, 30.4, 29.8, 29.7, 29.5, 26.17, 22.74, 14.2 ppm. **HRMS** (ESI$^+$): Calculated for $C_{47}H_{83}IN_3O_4$: 880.5422 [M+H]$^+$. Found: 880.5454 [M+H]$^+$. **m. p.** 115.4-117.7 ºC.

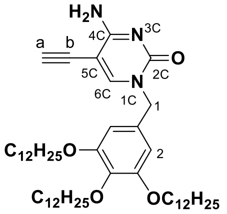

C0. Following *Standard Procedure B*, to a solution of **C0.1** (500.0 mg, 0.6 mmol), $Pd(Ph_3)_2)Cl_2$ (7.7 mg, 0.01 mmol), and CuI (1.1 mg, 0.01 mmol) in THF/Et_3N (5 mL), TMSA (1.4 mL, 2.3 mmol) was added and the mixture stirred at 40 ºC overnight. Once the reaction was completed, the solvent was evaporated, the resulting crude was suspended in THF (10 mL) and TBAF·$3H_2O$ (625.0 mg, 0.6 mmol) was added. After completion the solvent was evaporated and the resulting residue was purified by column chromatography eluted with $CHCl_3$:MeOH (50:1), affording **C0** as a pale solid (340.0 mg, 77%). **^{1}H-NMR** (300 MHz, $CDCl_3$): δ = 7.47 (s, 1H, H^{6C}), 6.87 (s broad, 1H, C^{4}NH-*H*), 6.49 (s, 2H, H^2), 5.67 (s broad, 1H, C^{4}NH-*H*), 4.87 (s, 2H, N^{1}CH_2), 3.93 (td, J = 6.5, 2.2 Hz, 6H, OCH_2), 3.32 (s, 1H, C≡C*H*), 1.89-1.52 (m, 6H, OCH_2CH_2), 1.28 (m, 54H, $OCH_2CH_2(CH_2)_9CH_3$), 1.03-0.67 (m, 9H, $O(CH_2)_{11}CH_3$) ppm. **^{13}C-NMR** (75 MHz, $CDCl_3$): δ = 165.0, 155.1, 153.6, 148.3, 138.4, 130.6, 107.1, 90.2, 84.0, 77.3, 75.0, 73.4, 69.3, 52.7, 32.0, 30.4, 29.8, 29.7, 29.5, 26.2, 22.7, 14.2 ppm. **HRMS** (ESI$^+$): Calculated for $C_{49}H_{84}N_3O_4$: 778.6456 [M+H]$^+$. Found: 778.6464 [M+H]$^+$. **m. p.** 94.2-95.9 ºC.

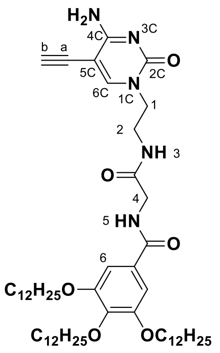

C2. To a suspension of **C2.1**[4] (270.0 mg, 1.0 mmol) in CH_2Cl_2 (26 mL), TFA (4.0 mL, 51.4 mmol) was added. The resulting solution was stirred at room temperature for 3 h, then TFA was removed by coevaporation with CH_2Cl_2 under reduced pressure. The resulting crude was re-dissolved in dry DMF and stirred at room temperature for 20 minutes, then cooled to 0 ºC. Meanwhile, to a solution of the acid **W2**[3] (710.0 mg, 1.0 mmol) in CH_2Cl_2 at 0 ºC, EDC (267.2 mg, 1.9 mmol), HOBt (331.2 mg, 2.5 mmol) and DIPEA (0.3 mL, 1.9 mmol) were added. The solution was stirred at room temperature for one hour, the solvent was removed under reduced pressure and the crude re-dissolved with DMF and a few drops of a $CHCl_3$:MeOH (10:1) mixture. This solution was added to the solution at 0 ºC of the deprotected amine, and then stirred at room temperature overnight. Once the reaction was completed, the solution was washed with HCl (0.1 M), $NaHCO_3$ (sat) and NaCl (sat). The phases were separated and the organic layer was dried with $MgSO_4$ and the solvent evaporated under reduced pressure. The crude product was purified by column chromatography using a 10:1 $CHCl_3$:MeOH mixture as eluent, affording a white solid (89.1mg, 50%). **^{1}H-NMR** (300 MHz, $CDCl_3$): δ = 8.71 (s broad, 1H, NH^5), 7.81 (s broad,1H, NH^3), 7.46 (s, 1H, H^{6C}), 7.08 (s, 2H, H^6), 4.21 (s broad, 2H, C$H_2{}^4$), 4.19-3.87 (m, 8H, OCH_2, C$H_2{}^2$), 3.59 (s broad, 2H, C$H_2{}^1$), 3.25 (s, 1H, C≡C*H*), 1.79-1.67 (m, 6H, OCH_2CH_2), 1.45-1.13 (m, 54H, $OCH_2CH_2(CH_2)_9CH_3$), 0.88-0.84 (m, 9H, $O(CH_2)_{11}CH_3$) ppm. **^{13}C-NMR** (75 MHz, $CDCl_3$): δ = 170.6, 168.1, 165.0, 155.6, 153.2, 150.5, 141.7, 128.7, 106.3, 90.0, 83.9, 75.3, 73.7, 69.6, 50.2, 43.8, 38.1, 32.1, 30.5,

29.91, 29.87, 29.85, 29.82, 29.75, 29.6, 29.54, 29.52, 26.29, 26.25, 22.9, 14.7. **MS** (ESI$^+$): Calculated for $C_{53}H_{90}N_5O_6$ [M+H]$^+$: 892.68; found: 892.66 [M+H].

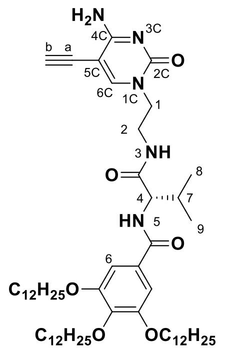

C2*. To a suspension of **C2.1** (250.0 mg, 0.9 mmol) in CH_2Cl_2 (15 mL), TFA was added (3.0 mL, 0.04 mmol), the resulting solution was stirred at room temperature for 3.5 h. Then, TFA was removed by coevaporation with CH_2Cl_2. The resulting solid was re-dissolved in CH_2Cl_2 (25 mL), then Et_3N (276.0 µL, 2.0 mmol) was added and the solution was stirred for 15 minutes. In another flask to a solution of the acid **W2*** (465.0 mg, 0.6 mmol) in dry CH_2Cl_2 (25 mL) at 0 ºC, HOBt (162.0 mg, 1.2 mmol) and EDC (230.0 mg, 1.2 mmol) were added. The mixture was stirred for 15 minutes, then, the solution of the deprotected amine was added. The mixture was stirred at room temperature overnight. Once the reaction was completed, the solution was diluted with CH_2Cl_2 and then washed with HCl (0.1 N), $NaHCO_3$ (sat) and NaCl (sat). The phases were separated, and the organic layer was dried with Na_2SO_4 and then the solvent was evaporated. The crude product was purified by column chromatography, eluted with $CHCl_3$: MeCN (30:1) obtained **C2*** as a white solid (404.0 mg, 48%). **^{1}H-NMR** (300 MHz, $CDCl_3$) δ = 9.01 (m, 1H, N^3H), 7.87 (s broad, 1H, NH-*H*), 7.27 (s, 1H, CH^6), 7.05 (s, 2H, H^6), 6.94 (d, J = 9.3 Hz, 1H, N^5H), 5.56 (broad, 1H, NH-*H*), 5.09 (dd, J = 6.9, 9.3 Hz, 1H, H^4), 4.17-3.92 (m, 2H, $CH_2{}^2$), 3.60-3.58 (m, 2H, N^1CH_2) 4.02 (m, 6H, OCH_2), 2.96 (s, 1H, C≡C*H*), 2.15 (m, 1H, H^7), 1.88-1.64 (m, 6H, OCH_2CH_2), 1.54-1.19 (m, 54H, $OCH_2CH_2(CH_2)_9CH_3$), 1.04 (d, J = 8.2 Hz, 3H, $CH_3{}^8$), 1.02 (d, J = 8.2 Hz, 3H, $CH_3{}^9$), 0.89 (m, 9H, O $(CH_2)_{11}CH_3$) ppm. **^{13}C-NMR** (75 MHz, $CDCl_3$) δ = 172.8, 167.5, 164.9, 155.5, 153. 3, 150.5, 141.8, 129.3, 106.5, 89.8, 83.4, 77.3, 75.4, 73.8, 69.8, 58.7, 49.8, 37.20, 32.26, 32.08, 32.07, 30.5, 29.89, 29.85, 29.80, 29.74, 29.61, 29.59, 29.53, 29.51, 26.28, 26.25, 22.8, 19.5, 18.65, 14.24 ppm. **MS** (ESI$^+$): Calculated for $C_{56}H_{95}N_5O_6$ [M+H]$^+$: 934.40; found: 935.75 [M+H]$^+$. **[α]$^D{}_{20}$** = -1.21 (c 1, $CHCl_3$)

Synthetic procedures and characterization data for the new G-C final monomers (GC0, GC2 and GC2*)

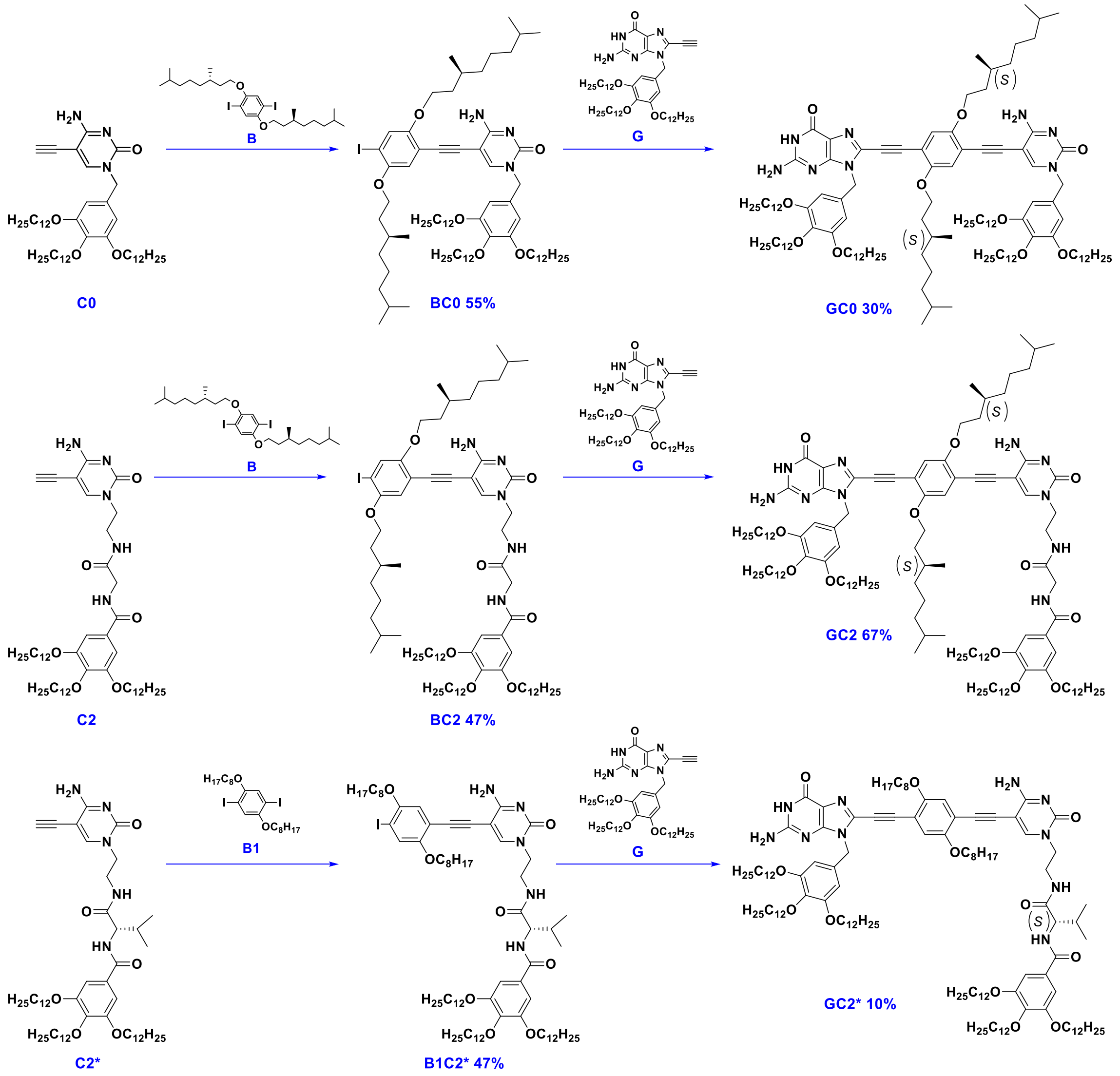

Scheme S3.

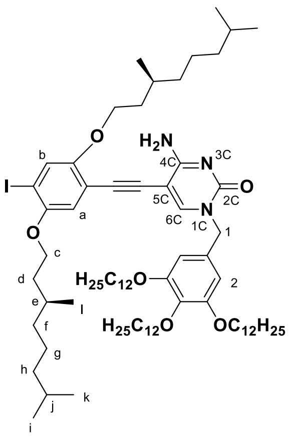

BC0. Following *Standard Procedure C*, **B**[5] (1.0 g, 1.56 mmol), **C0** (403.0 mg, 0.51 mmol), $Pd(Ph_3)_2Cl_2$ (6.8 mg, 0.01 mmol) and CuI (0.9 mg, 0.005 mmol) were dissolved in a THF:Et_3N mixture (15 mL). the resulting mixture was stirred at 40ºC overnight. Once the reaction was completed, the solvent was removed under reduced pressure. The resulting product was purified by column chromatography, using as eluent a $CHCl_3$:MeOH 50:1 mixture. The product was obtained as a colorless oil in 55 % yield. (345 mg, 55%).**^{1}H-NMR** (300 MHz, $CDCl_3$): δ =7.41 (s, 1H, H^{6C}), 7.28 (s, 1H, 1H^b), 6.85 (s, 1H, H^a), 6.78 (s broad, 1H, NH-*H*), 6.49 (s, 2H, H^2), 6.16 (bs, 1H, NH-*H*), 4.89 (s, 2H, $CH_2{}^2$), 4.04-3.89 (m, 10H, $OCH_2(CH_2)_{10}CH_3$, $CH_2{}^c$), 1.95–1.69 (m, 10H, $OCH_2CH_2(CH_2)_9CH_3$, $CH_2{}^d$), 1.67-1.52 (m, 2H, H_j), 1.46-1.08 (m, 70H, $OCH_2CH_2(CH_2)_9CH_3$, $CH_2{}^{f,g,h}$, $H^{e,j}$), 1.01–0.76 (m, 27H,

$O(CH_2)_{11}CH_3$, $CH_3^{k,l,l}$). **HRMS** (ESI^+): Calculated for $C_{75}H_{127}IN_3O_6$: 1292.8764 $[M+H]^+$. Found: 1292.8772 $[M+H]^+$.

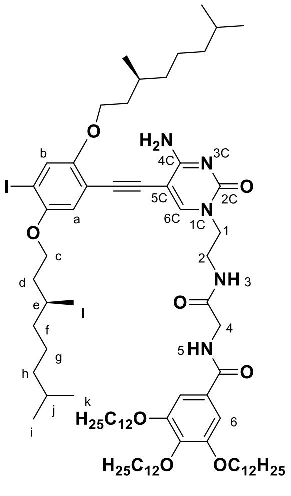

BC2. Following *Standard Procedure C*, **C2** (100.0 mg, 0.11 mmol), **B**[5] (115.3 mg, 0.34 mmol), $Pd(Ph_3)_2Cl_2$ (1.57mg, 0.002 mmol) and CuI (0.2 mg, 0.001 mmol) were dissolved in a THF:Et_3N mixture. The solution was stirred at 40ºC overnight. The solvent was then removed under reduced pressure, and the resulting product was purified by column chromatography, using as eluent a $CHCl_3$:MeOH 100:1 solvent mixture. The product was obtained as a colorless oil in 47% yield. (74.1 mg, 47%). **^{1}H-NMR** (300 MHz, $CDCl_3$): δ = 8.32 (s broad, 1H, NH^5), 7.44 (s, 1H, H^{6C}), 7.09 (s, 1H, H^b), 7.10 (s, 1H, H^a), 7.06 (s, 2H, H^6), 7.01 (s broad, 1H, NH-*H*), 6.75 (s, 1H, NH^3), 6.25 (s broad, 1H, NH-*H*), 4.22 (qd, *J* = 16.3, 5.7 Hz, 2H, CH_2^4), 4.02-3.89 (m, 10H, $OCH_2(CH_2)_{10}CH_3$, CH_2^c, CH_2^2), 3.76-3.60 (m, 4H, CH_2^d), 1.80-1.67 (m, 6H, $OCH_2CH_2(CH_2)_9CH_3$), 1.65–1.51 (m, 8H, H^e, CH_2^f, CH_2^1), 1.51–0.99 (m, 66H, $OCH_2CH_2(CH_2)_9CH_3$, $CH_2^{g,h}$, H^j), 0.86 (m, 27H, $O(CH_2)_{11}CH_3$, $CH_3^{l,l,k}$). **HRMS** (MALDI+): Calculated for $C_{79}H_{132}N_5O_8I$: 1406.9193 $[M+H]^+$. Found: 1406.9187 $[M+H]^+$.

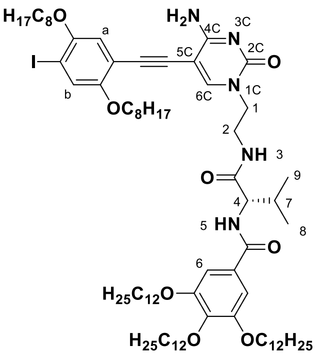

B1C2*. Following *Standard Procedure C* for a Sonogashira coupling, **C2*** (200.0 mg, 0.214 mmol), **B1**[6] (627.2 mg, 1.07 mmol), $Pd(Ph_3)_2Cl_2$ (3.0 mg, 0.004 mmol) and CuI (0.4 mg, 0.0002 mmol) were dissolven in a THF:Et_3N mixture (10 mL). The resulting solution was stirred at r.t. overnight, and then heated at 50ºC for 1h. Once the reaction was completed, the solvent was evaporated under reduced pressure. The residue was then purified by column chromatography, using as eluent a $CHCl_3$:MeOH (100:1) mixture. The product was obtained as a white solid in 47 % yield. (140.0 mg, 47%). **^{1}H-NMR** (300 MHz, $CDCl_3$) δ = 9.01 (m, 1H, N^3H), 7.87 (s broad, 1H, NH-*H*), 7.27 (s, 1H, CH^6), 7.20 (s, 1H, H^b), 7.05 (s, 2H, H^6), 7.03 (s, 2H, H^a), 6.94 (d, *J* = 9.3 Hz, 1H, N^5H), 5.56 (broad, 1H, NH-*H*), 5.09 (dd, *J* = 6.9, 9.3 Hz, 1H, H^4), 4.17-3.92 (m, 2H, CH_2^2), 3.60-3.58 (m, 2H, N^1CH_2) 4.02 (m, 10H, OCH_2), 2.15 (m, 1H, H^7), 1.88-1.64 (m, 10H, OCH_2CH_2), 1.54-1.19 (m, 74H, $OCH_2CH_2(CH_2)_9CH_3$, $OCH_2CH_2(CH_2)_5CH_3$), 1.04 (d, *J* = 8.2 Hz, 3H, CH_3^8), 1.02 (d, *J* = 8.2 Hz, 3H, CH_3^9), 0.89 (m, 15H, $O(CH_2)_{11}CH_3$, $O(CH_2)_7CH_3$) ppm.

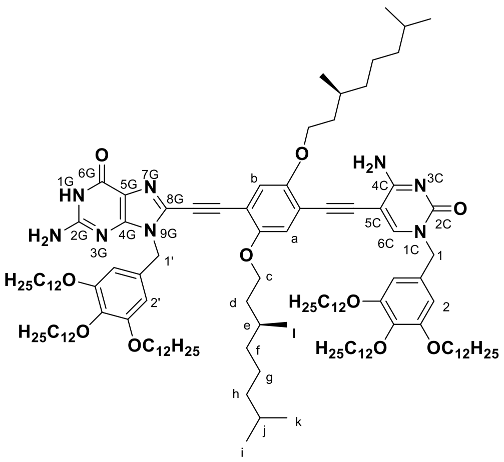

GC0. Following *Standard Procedure C* for a Sonogashira coupling, **BC0** (100.0 mg, 0.082 mmol), **G**[4] (80.0 mg, 0.097 mmol), $Pd(Ph_3)_4$ (2.226 mg, 0.002 mmol) and CuI (0.2 mg, 0.001 mmol) were dissolved in a $THF{:}Et_3N$ mixture (3 mL). The mixture was stirred at 40ºC for 12h. Once the reaction was completed, the solvent was evaporated under reduced pressure. The crude product was purified by column chromatography, using as eluent a $CHCl_3$:MeOH 100:1 mixture. The product was obtained as a yellow solid in 30% yield. (48.9 mg, 30%). **^{1}H-NMR** (300 MHz, $CDCl_3$): δ =7.64 (s, 1H, NH-*H*), 7.41 (s, 1H, H^{6C}), 7.13 (s, 1H, H^b), 7.01 (s, 1H, H^a), 6.79 (s, 2H, H^{2}), 6.53 (s, 2H, H^{2}), 5.38 (s, 3H, $CH_2^{1'}$), 4.94 (s, 2H, CH_2^{1}), 4.21 – 3.82 (m, 20H, $OCH_2(CH_2)_{10}CH_3$, $CH_2^{c,d}$), 1.94-1.01 (m, 136H, $OCH_2(CH_2)_{10}CH_3$), $CH_2^{,f,g,h}$, H^e, H^j), 1.01 – 0.75 (m, 36H, $O(CH_2)_{11}CH_3$, $CH_3^{i,k,l}$). **HRMS** (MALDI+): Calculated for $C_{127}H_{211}N_9O_{11}Na$: 2062.6153 $[M+Na]^+$. Found: 2062.6219 $[M+Na]^+$.

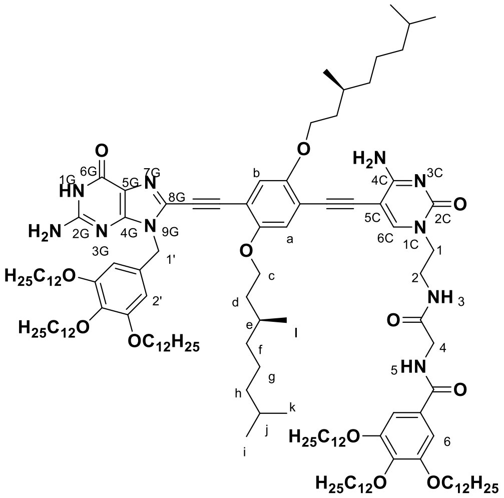

GC2. Following *Standard Procedure C* for a Sonogashira coupling **BC2** (100.0 mg, 0.071 mmol), **G**[4] (70.0 mg, 0.085 mmol), $Pd(Ph_3)_4$ (1.15 mg, 0.001 mmol) and CuI (0.1 mg, 0.0007 mmol) were dissolved in a $THF{:}Et_3N$ mixture (3 mL) and stirred at 40ºc overnight. Once the reaction was completed, the solvent was removed under reduced pressure. The residue was then purified by column chromatography, using as eluent a $CHCl_3$:MeOH 100:1 mixture. The product was obtained as a yellow solid in 67 %yield. (100.5 mg, 67%). **^{1}H-NMR** (300 MHz, $CDCl_3$): δ = 7.98 (s broad, 1H NH^5), 7.75 (s broad, 1H, NH^3), 7.51 (s, 1H, H^{6C}), 7.13 (s, 1H, H^b), 7.03 (s, 1H, H^a), 6.99 (s, 2H, H^6), 6.80 (s, 2H, $H^{2'}$), 5.39 (s, 2H, $CH_2^{1'}$), 4.34–3.94 (m, 20H, $OCH_2(CH_2)_{10}CH_3$, $CH_2^{c,d}$), 3.89-3.70 (m, 6H, $CH_2^{4,2,1}$), 1.74-1.50 (m, 12H, $CH_2^{,f,g,h}$), 1.49-0.96 (m, 112 H, $OCH_2CH_2(CH_2)_9CH_3$, H^e, H^j), 0.87 (m, 36H, $O(CH_2)_{11}CH_3$, $CH_3^{i,k,l}$) ppm. **HRMS** (ESI^+): Calculated for $C_{129}H_{215}N_{10}O_{12}$: 2097.6548 $[M+H]^+$. Found: 2097.6566 $[M+H]^+$.

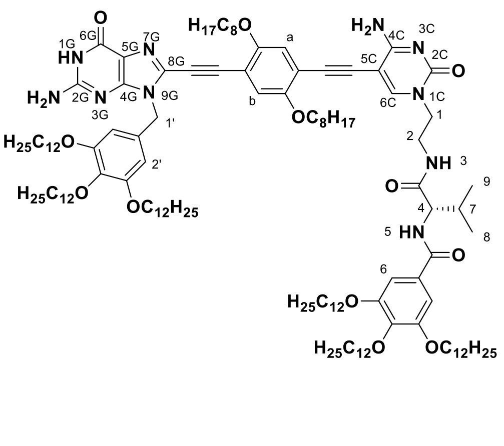

GC2*. Following *Standard Procedure C* for a Sonogashira coupling, **B1C2*** (101.7 mg, 0.071 mmol, **G**[4] (72 mg, 0.087 mmol), $Pd(Ph_3)_4$ (1.15 mg, 0.001 mmol) and CuI (0.13 mg, 0.0007 mmol) in THF. Then a few drops of $^iPr_2NH$ were added, and the solution was stirred at r.t. overnight. Once the reaction was completed, the mixture was filtered over a celite plug and washed with $CHCl_3$:MeOH 10:1. The solvent was then evaporated under reduced pressure and the residue was

purified by column chromatography, using as eluent $CHCl_3$:MeOH 50:1. The product was obtained as a yellow solid in 10 % yield. (16 mg, 10 %). %). **^{1}H-NMR** (300 MHz, $CDCl_3$, TFA 1%) δ = 7.96 (s, 1H, H^{6C}), 7.54 (s, 1H, H^b), 7.12 (s, 1H, H^a), 7.04 (s, 2H, H^6), 7.00 (s broad, 1H, NH^3), 6.80 (s, 2H, $H^{2'}$), 5.40 (s, 2H, C$H_2{}^{1'}$), 4.23–3.93 (m, 17H, OC$H_2$$(CH_2)_{10}CH_3$, H^4, OC$H_2$$(CH_2)_6CH_3$), 3.90-3.71 (m, 4H, C$H_2{}^2$, C$H_2{}^1$), 1.85-1.72 (m, 16H, OCH_2C$H_2$$(CH_2)_9CH_3$, OCH_2C$H_2$$(CH_2)_5CH_3$), 1.51-1.00 (m, 129H, OCH_2CH_2(C$H_2$$)_9CH_3$, OCH_2CH_2(C$H_2$$)_5CH_3$, H^7), 1.11 – 0.73 (m, 30H, $O(CH_2)_{11}$CH_3, $O(CH_2)_7$CH_3, C$H_3{}^8$, C$H_3{}^9$) ppm. **HRMS** (ESI^+): Calculated for $C_{128}H_{212}N_{10}O_{12}$: 2083.6391 $[M+H]^+$. Found: 2083.6391 $[M+H]^+$.

IR characterization

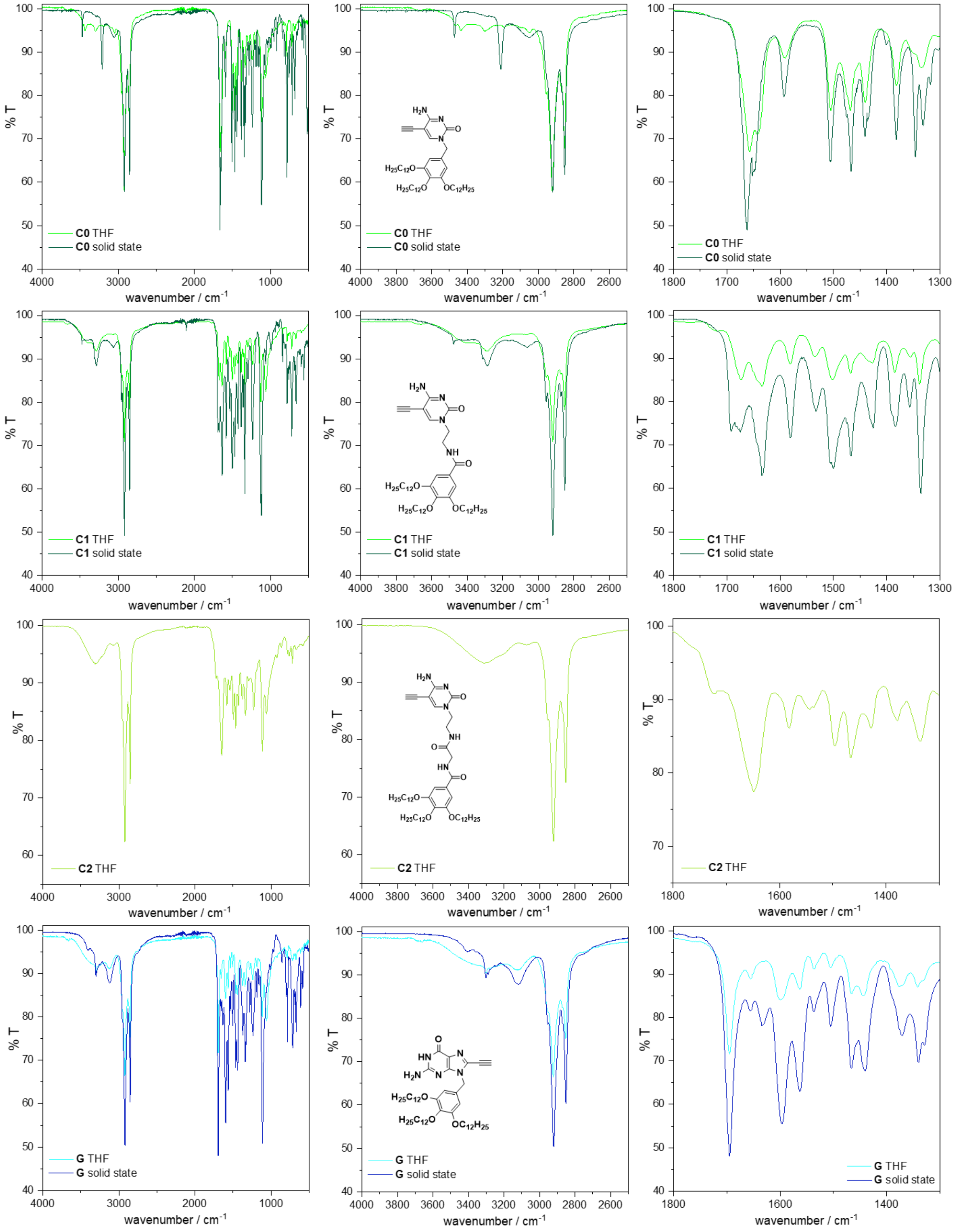

Figure S0A. IR spectra of the nucleobases **C0**, **C1**, **C2** and **G** in the solid state compared to a THF solution at 1.0·10^{-3} M.

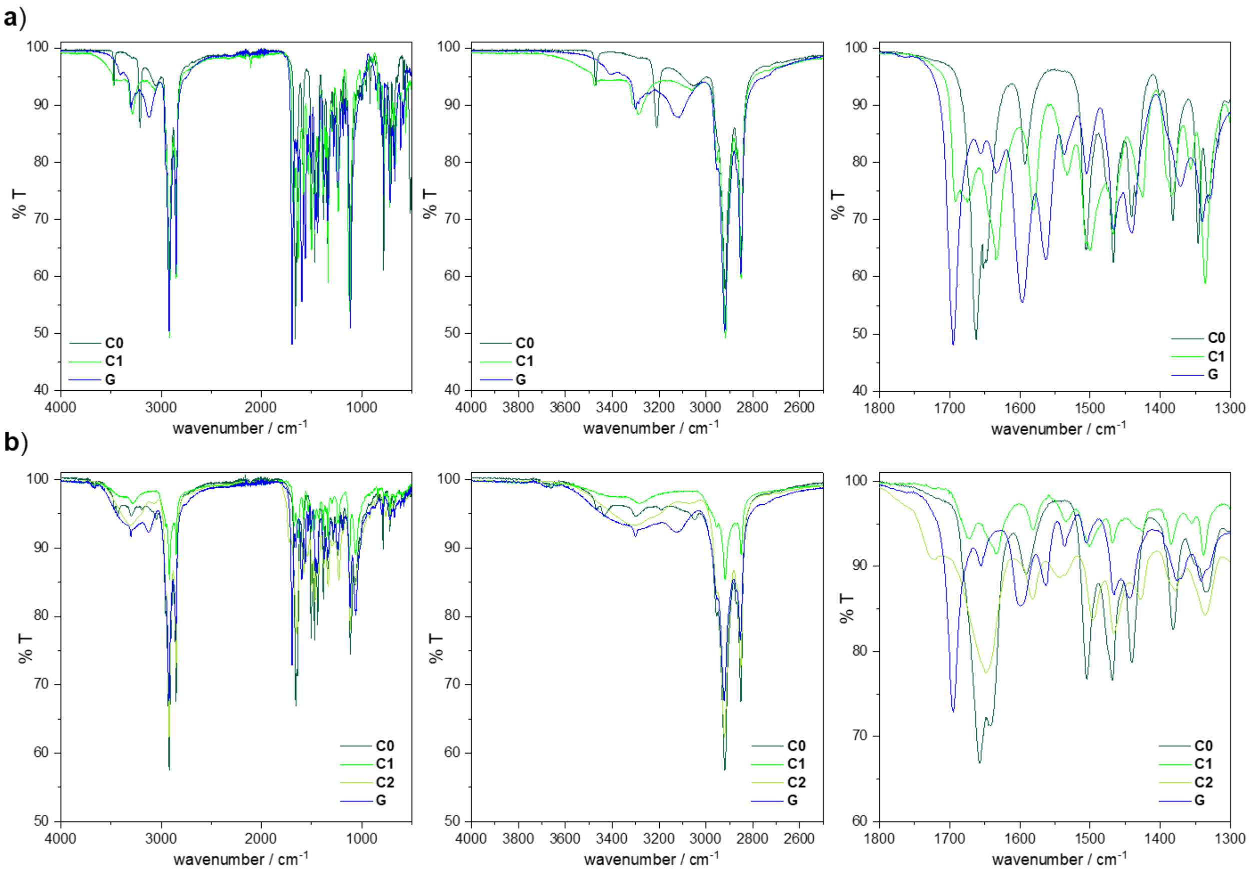

Figure S0B. Comparison of the IR spectra of the nucleobases **C0, C1, C2 and G** in (**a**) the solid state and (**b**) a THF solution at $1.0 \cdot 10^{-3}$ M.

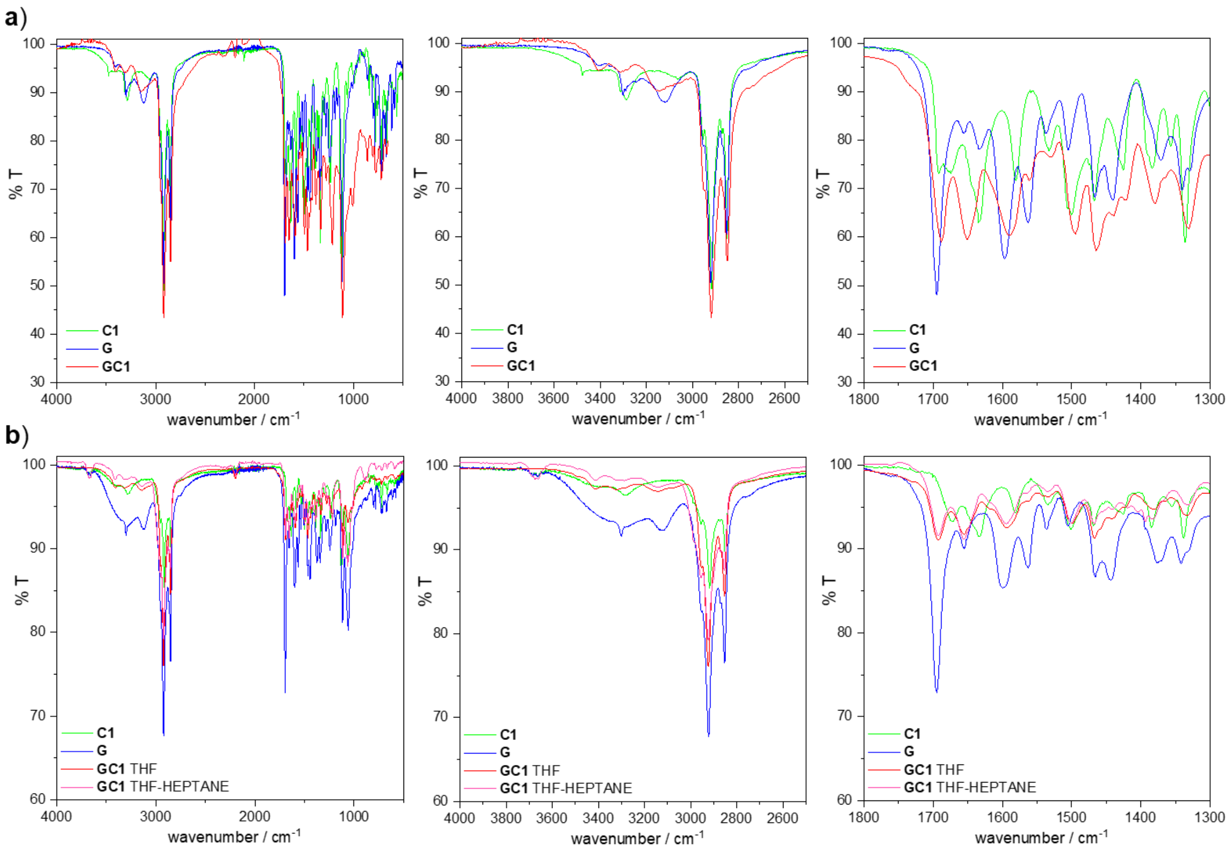

Figure S0C. Comparison of the IR spectra of **C1**, **G** and **GC1** in (**a**) the solid state and (**b**) THF and THF-heptane mixture (1:99) at $1.0{\cdot}10^{-3}$ M.

S1. UV-vis and emission spectra in diverse solvents

Preliminary UV-vis and emission spectroscopy studies were carried out in solvents of different polarity (DMAC, THF, chlorobenzene and toluene) at a concentration of $5.0 \cdot 10^{-5}$ M at 293 K (Figure S1). These solutions showed different aggregation states for the 3 studied monomers (**GC0**-**GC2**) at room temperature as a function of the solvent environment. The changes in the UV-vis spectra are subtler than the changes observed by other techniques such as emission or CD (see below). In the solutions prepared in the more polar solvent (DMAC), which strongly competes for H-bonding (like DMSO or DMF), two absorption maxima were observed at *ca.* 394 nm and 413 nm and a single emission maximum at *ca.* 456 nm, which is characteristic of the monomeric state. In contrast, in apolar environments, such as chlorobenzene and toluene, the absorption maxima of the 3 monomers were red-shifted to *ca.* 400 nm and 422 nm, while the emission intensity decreased and shifted to a new maximum at *ca.* 525 nm, which indicates, according to our previous work,[7-8] complete cyclic tetramer formation for the 3 monomers. Solvents of intermediate polarity, like THF, allowed us to monitor the monomer (GC)-cyclic tetramer ($c\mathrm{GC}_4$) equilibria at concentrations between 10^{-2} M to 10^{-5} M (please see our previous work). In this solvent, the absorption spectra showed the same maxima observed in DMAC, although it differed in intensity, since the second maximum at 413 nm had a lower intensity than the maximum at 394 nm. In the emission spectra, the characteristic monomer maximum is still present, but a shoulder around 525 nm was also clearly detected. This is in agreement with the coexistence of monomer and cyclic species, which is also in line with the ^{1}H-NMR experiments carried out in THF in similar concentration and temperature conditions (see below). Interestingly, the relative intensity of the 525 nm emission shoulder increased in the order **GC2** > **GC0** > **GC1**, which already discloses preliminary stability trends.

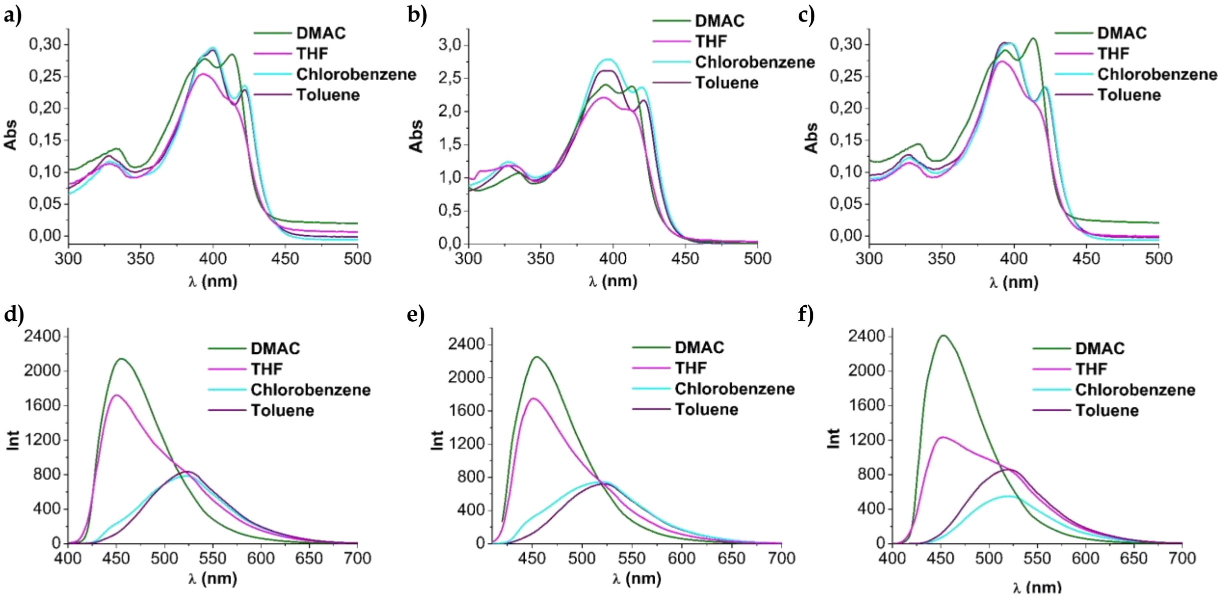

Figure S1. UV-vis (**a-c**) and emission (**d-f**) spectra of (**a,d**) **GC0**, (**b,e**) **GC1** and (**c,f**) **GC2** at $5.0 \cdot 10^{-5}$ M in different solvents.

S2. Monomer-Cyclic Tetramer Equilibria Studied in THF

^{1}H NMR and CD spectroscopies are even better techniques to monitor the monomer (GC)-cyclic tetramer (c(GC)$_4$) equilibria at concentrations between 10^{-2} M and 10^{-4} M (^{1}H NMR) or 10^{-3} M and 10^{-6} M (CD) in a solvent of intermediate polarity like THF. To this end, several temperature- and concentration dependent experiments were carried out with the three CD-active monomers: **GC0**, **GC1** and **GC2**.

S2.1. Temperature-dependent Experiments

As shown in Figure S2A, ^{1}H-NMR temperature-dependent experiments were first carried out in THF-d_8 in order to calculate the molar fraction of the two different species (GC and c(GC)$_4$) at different temperatures and to evaluate the thermodynamic stability of each cyclic tetramer. In all cases, the typical H-bonded G-amide and C-amine proton signals were detected at around 13.5 and 10.0 ppm, and an *all or nothing* monomer-cyclic tetramer equilibrium was observed, where the two species were detected in slow exchange in the NMR timescale, as it is usual with this kind of GC monomers.[7-8]

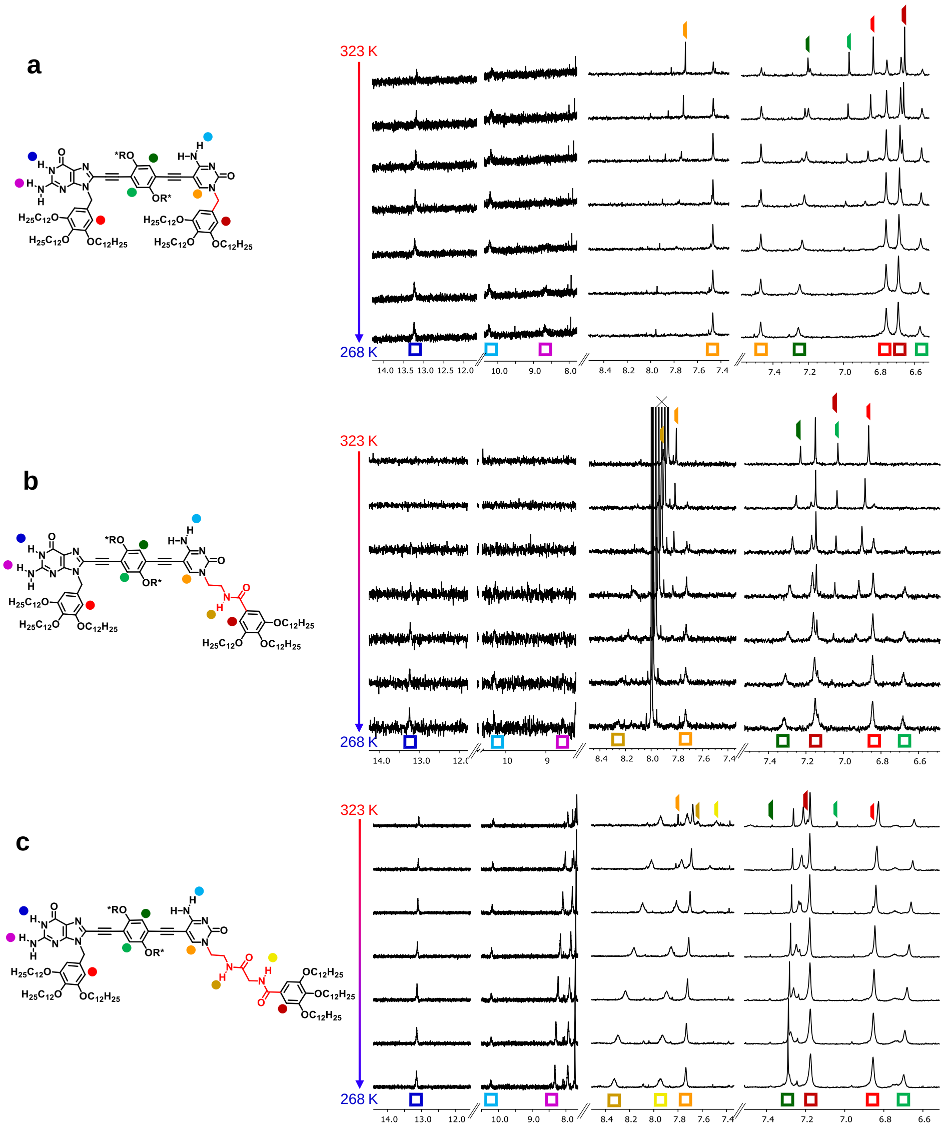

Figure S2A. Three different ^{1}H NMR regions in temperature-dependent experiments at 1.0·10^{-4} M in THF-d$_8$ for (**a**) **GC0**, (**b**) **GC1** and (**c**) **GC2**.

These NMR experiments provided valuable information about the presence of H-bonding interactions between complementary bases and about the formation of macrocyclic species. However, and as mentioned in the main text, CD spectroscopy turns out to be also very informative to assess the formation of cyclic species along our whole studies with dinucleoside monomers.[7-9] This kind of molecules can alternate between different conformations, by rotation around the σ-bonds present along their rigid π-conjugated skeleton, that dispose the nucleobase Watson-Crick edges either at the same side or at opposite sides of the molecule. Only when these monomers form a cyclic structure, this degree of freedom is lost and the Watson-Crick edges in the terminal nucleobases are locked pointing to the same side. Apparently, this conformational "freezing" of the monomer skeleton upon cyclization allows the chiral riboses to interact and transfer their chiral information to the π-conjugated backbone. In this way, a Cotton effect appears upon cyclization that matches the NMR trends in the same conditions and that can be used to monitor and quantify cyclic tetramer formation in a complementary manner and within a more dilute concentration window, whereby monomer or any other non-cyclic oligomers are usually CD-inactive.

Hence, temperature-dependent absorption and CD experiments for **GC0-GC2** were also carried out in the same conditions of the ^{1}H NMR experiments (THF, $1.0{\cdot}10^{-4}$ M), as shown in Figure S2B, so as to monitor the GC- c(GC)$_4$ equilibrium. A similar stability trend as that found in the ^{1}H-NMR experiments was observed. Whereas c(**GC1**)$_4$ was fully dissociated at high temperatures, c(**GC0**)$_4$ and, particularly, c(**GC2**)$_4$ still displayed the characteristic cyclic tetramer CD signals even at the highest temperatures.

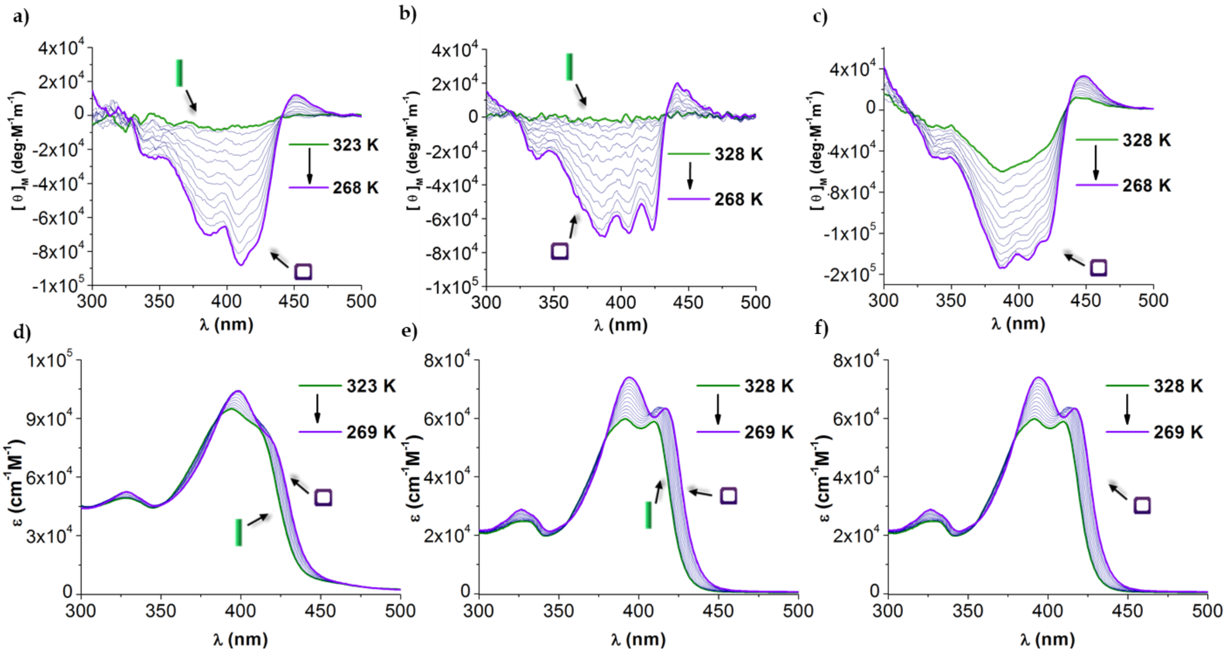

Figure S2B. Temperature-dependent (**a-c**) CD and (**d-f**) absorption spectra for (**a,d**) **GC0**, (**b,e**) **GC1** and (**c,f**) **GC2** at $1.0{\cdot}10^{-4}$ M in THF.

S2.2. Concentration-dependent Experiments

Dilution experiments were also performed with **GC0-GC2** in THF-*d*$_8$ within the $2.0{\cdot}10^{-3}$-$1.0{\cdot}10^{-4}$ M range at a constant 298 K temperature (Figure S2C). Higher concentrations resulted in incomplete solubility, while lower concentrations were not optimal for signal integration, even with a large number of scans. As shown in Figure S2C, and in analogy to the previous experiments, the $c(\mathrm{GC})_4$ species is formed almost quantitatively at high concentrations and is dissociated into GC monomer as the concentration is reduced. Again, only two sets of signals in slow exchange at the NMR timescale and with almost identical shape and position, were observed during these dilution experiments for the 3 molecules. At the lowest $1.0{\cdot}10^{-4}$ M concentration, the signals corresponding to **GC1** molecules in monomer and cyclic tetramer species are at an approximately 1:1 ratio ($\chi_T = 0.5$). In contrast, for **GC0** the monomer concentration in the same conditions is far lower, whereas for **GC2** the monomer could not even be detected. This confirms the same stability trend already noted in the temperature-dependent experiments: *c*(**GC2**)$_4$ > *c*(**GC0**)$_4$ > *c*(**GC2**)$_4$.

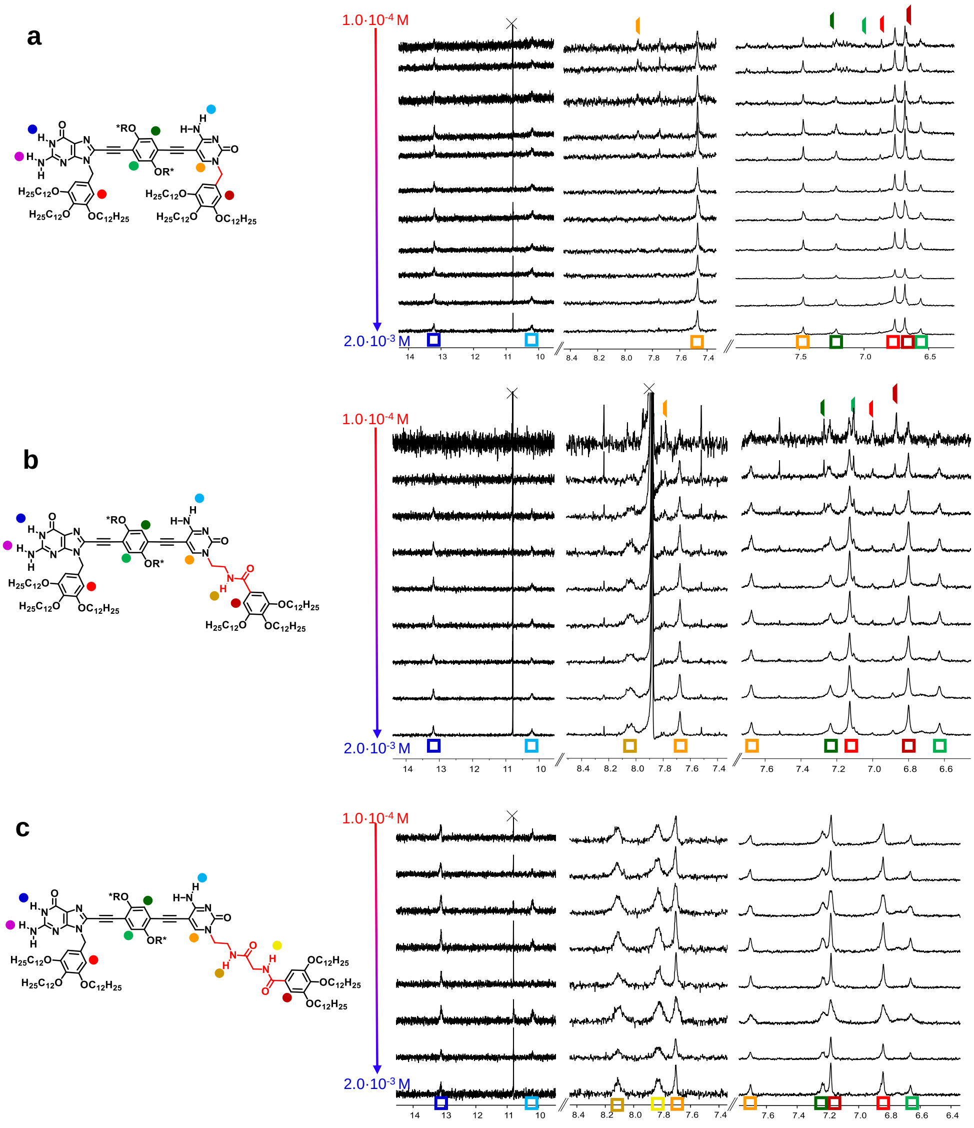

Figure S2C. Three different ^{1}H NMR regions in dilution experiments in THF-d$_8$ at 298 K of (**a**) **GC0**, (**b**) **GC1** and (**c**) **GC2**.

Dilution experiments monitored by CD were next performed in a concentration range that overlapped the ^{1}H NMR experiments and that could be extended to *ca.* $5{\cdot}10^{-6}$ M (Figure S2D). The same stability trend was again observed: *c*(**GC2**)$_4$ > *c*(**GC0**)$_4$ > *c*(**GC2**)$_4$.

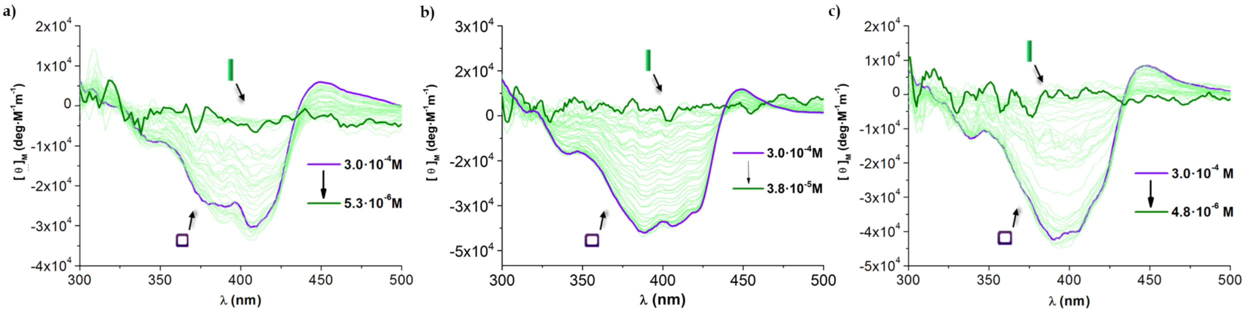

Figure S2D. CD dilution experiments in THF of (**a**) **GC0**, (**b**) **GC1** and (**c**) **GC2**.

S2.3. Solvent-dependent Experiments

Different ^{1}H NMR spectra at gradually increasing volume fractions of cyclohexane-d_{12} into THF-d_8 were recorded with the aim to see a change in the peripheral amide chemical shifts just upon polymerization. However, when *c*(**GC1**)$_4$ and *c*(**GC2**)$_4$ start to polymerize, their ^{1}H NMR signal become too broad and disappear before any H-bonding rearrangement can be detected (Figure S2E).

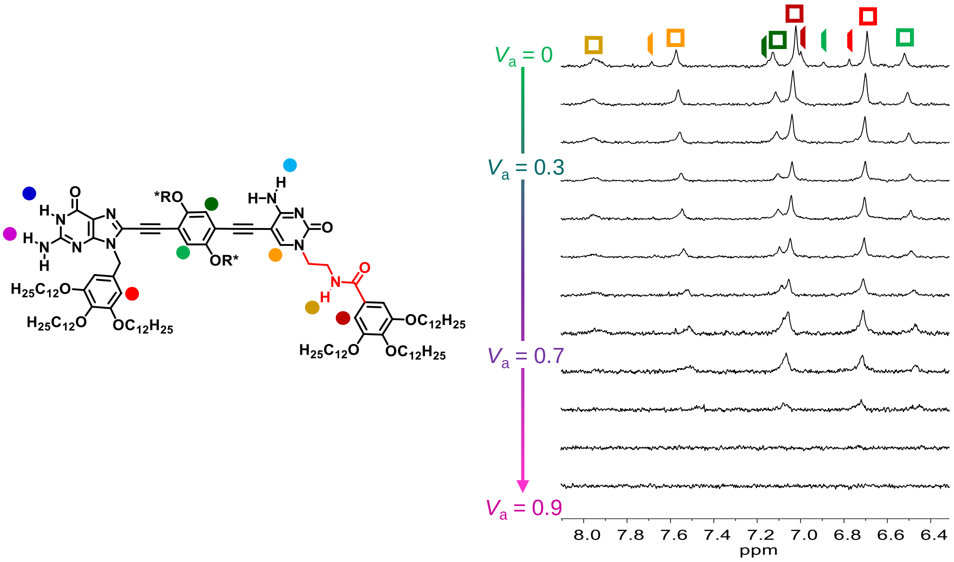

Figure S2E. Changes observed in the aromatic region of the ^{1}H NMR spectra of **GC1** by increasing the volume fraction of cyclohexane-d_{12} (V_a) in mixtures with THF-d_8 ([**GC1**] = $3.0{\cdot}10^{-4}$ M; T = 298 K.

S2.4. Quantitative Analysis of the Cyclotetramerization Process in THF

As shown above, ^{1}H NMR experiments allowed us to calculate the relative concentration of monomer and cyclic tetramer species by integration of several isolated C-H proton signals. This data was employed to determine the molar fraction of GC molecules associated as $c(GC)_4$ macrocycles (χ_T) as a function of temperature and concentration. On the other hand, since the *S*-chiral GC monomers are inactive in CD, this technique was much more useful than absorption or fluorescence spectroscopy to monitor the cyclotetramerization process and estimate as well χ_T, avoiding the interference with the monomer signals.

With the aim of contrasting the data obtained in the temperature-dependent experiments monitored by ^{1}H NMR and CD (Figures S2A and S2B, respectively), χ_T was plotted as a function of temperature at different concentrations (Figure S2F). The experiments measured by both techniques show a rather good correlation, despite different solvent sources (deuterated *vs* non-deuterated THF) were employed. The stability differences already noted in each of the experiments can be better appreciated in these plots: while *c*(**GC1**)$_4$ could be totally dissociated with temperature in THF at a 10^{-4} M concentration, the dissociation of *c*(**GC2**)$_4$ was very small, and *c*(**GC0**)$_4$ displayed an intermediate behaviour.

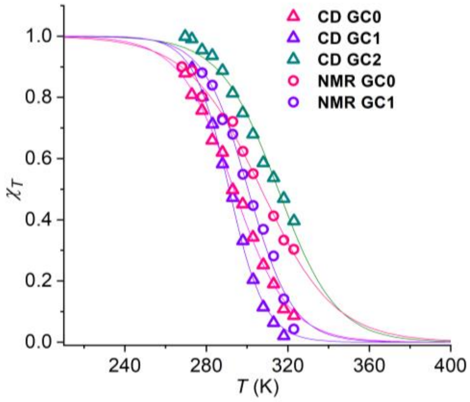

Figure S2F. Representation of the molar fraction of **GC0**-**GC2** molecules associated as cyclic tetramers (χ_T) as a function of the temperature, as determined by ^{1}H-NMR (Figure S2A) or CD (Figure S2B) experiments in THF.

In order to calculate the enthalpic (ΔH_T) and entropic (ΔS_T) changes associated to the cyclotetramerization process in THF, the tetramer and monomer concentrations were calculated from integration of the different ^{1}H-NMR temperature-dependent experiments (Figure S2A), and a van't Hoff representation of this data, shown in Figure S2G**,** yielded linear trends that could be fitted using the van't Hoff equation.

$$\ln(K) = -\frac{\Delta H}{\mathrm{R}}\left[\frac{1}{T}\right] + \frac{\Delta S}{\mathrm{R}} \qquad (1)$$

The calculated thermodynamic parameters are shown in Table S1. The only exception was *c*(**GC2**)$_4$, which did not show appreciable dissociation in these conditions and, therefore, prevented the integration of monomer signals. That is why the thermodynamic parameters K_T, ΔH_T and ΔS_T could not be calculated in THF for this compound, although a lower limit for K_T could be established.

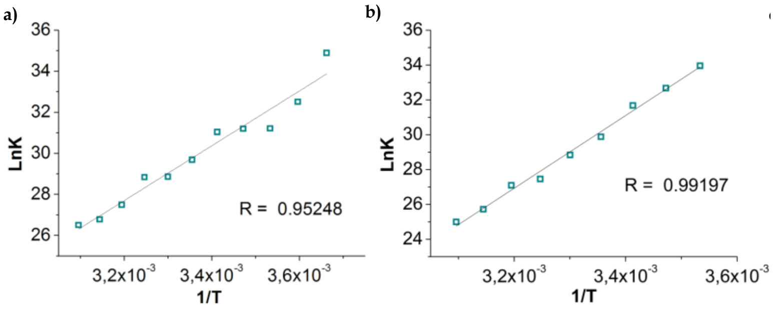

Figure S2G. Van't Hoff plots obtained for the macrocyclization process of (**a**) **GC0** and (**b**) **GC1**, extracted from the ^{1}H NMR temperature-dependent experiments in THF-d_8 at $1.0 \cdot 10^{-4}$ M shown in Figure S2A.

Table S1. Thermodynamic parameters calculated for the macrocyclization process of **GC0**-**GC2** obtained from the temperature-dependent ^{1}H NMR data in THF-d_8.

GC Compound	$K_{G:C}$[a] (M^{-1})	K_T[b] (M^{-3})	*EM*[c] (*M*)	ΔH_T (*kJ·mol*$^{-1}$)	ΔS_T (*J·mol*$^{-1}$·*K*$^{-1}$)
**GC0**	$0.55 \cdot 10^3$	$4.3 \cdot 10^{13}$	$4.7 \cdot 10^2$	-143.4	-215.1
**GC1**	$0.41 \cdot 10^3$	$3.4 \cdot 10^{12}$	$1.2 \cdot 10^2$	-173.4	-331.2
**GC2**	$0.77 \cdot 10^3$	$> \cdot 10^{14}$	$> 2.8 \cdot 10^2$	[4]	[4]

[a] $K_{G:C}$: Reference association constant calculated at 298 K in THF. [b] K_T: Cyclotetramerization constant at 298 K, K_T = [c(**GC**)$_4$]/[**GC**]^4. [c] *EM*: Effective Molarity, calculated as: $EM = K_T/(K_{G:C})^4$.[d] Could not be determined.

In order to compare the cooperativity of each cyclotetramerization process, the corresponding reference association constants for each G:C nucleobase pair ($K_{G:C}$)[10] were measured in separate titration experiments (see Figure S3A), and the effective molarity (*EM*) was calculated using the relationship $K_T = EM \cdot K_{G:C}{}^4$. From the data shown in Table S1, it is clear that, even if there are not important differences in $K_{G:C}$, the fact that each cycle is bound by 4 G:C interactions, and thereof $K_{G:C}$ is raised to the 4th power in the equation, can already explain the notable differences in cycle stability. The derived *EM* values, between 10^2 and 10^3 M, are, on the other hand, in line with our previous work.[11] Please note that the *EM* value of *c***GC1**$_4$ has been recalculated in this work by using the appropriate reference association constant between **G** and **C1** (see Figure S3A), instead of a previous $K_{G:C}$ value in THF obtained by us with lipophilic nucleosides ($K_{G:C} = 1.5 \cdot 10^3$ M^{-1}).[8] As a result, *EM* values are now considerably higher than those previously determined,[7] and in accordance with other G-C cyclic tetramers, as explained below.

It is also interesting to compare the stability of the *c*(**GC0**)$_4$–*c*(**GC2**)$_4$ macrocycles in comparison with related GC monomers published by us that differ in the absence of alkoxy substituentes in the phenylene central block and in the substituents at the nucleobases (i.e. bulky lipophilic ribose groups instead of the benzylic wedges; see our previous work).[7] $K_{G:C}$ values between those lipophilic guanosine and cytidine nucleosides in THF were slightly higher ($K_{G:C} = 1.5 \cdot 10^3$ M^{-1}), while the calculated *EM* values were around $4.3 \cdot 10^2$ M, very similar to those determined in this work. As a result, cyclotetramerization constants

On the other hand, the molar fraction of GC molecules associated as *c*(GC)$_4$ macrocycles (χ_T) could also be determined from the CD and ^{1}H-NMR dilution experiments (Figures S2C and S2D, respectively), and plotted as a function of the concentration, as shown in Figure S2H. The NMR and CD dilution data show again a nice overlap, and could be compared to simulated speciation curves produced with the *HySS*

software,[12] using K_T values that are in the range of the previously calculated values (Table S1). As shown in Figure S2H, a good agreement is seen between simulated and experimental data. These curves also illustrate graphically the *all-or-nothing* process observed in solution, where mostly GC monomer (green) and $c(GC)_4$ macrocycles (purple) are in equilibrium and the participation of small open oligomers (like dimer GC_2; in red) is insignificant.

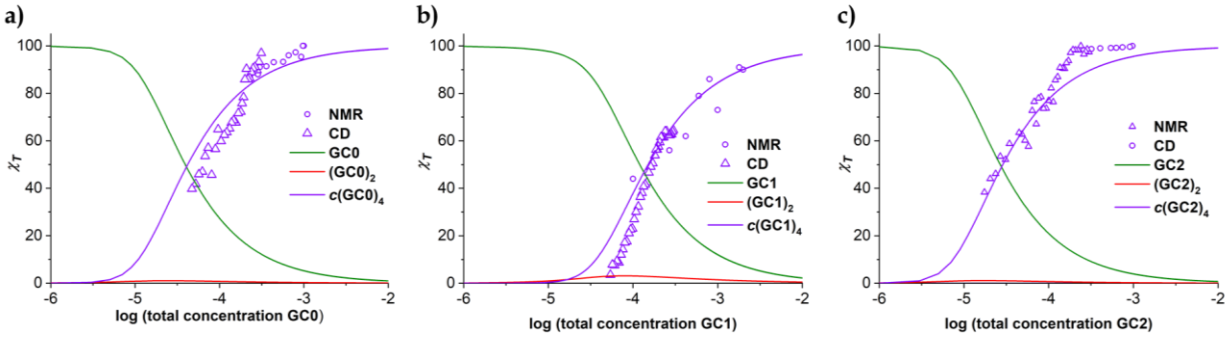

Figure S2H. Representation of the molar fraction of molecules associated as cyclic tetramers (χ_T), calculated by either ^{1}H-NMR (Figure S2C) or CD (Figure S2D), as a function of the overall concentration and simulated speciation curves (obtained with the Hyss software) of the main different species in solution for (**a**) **GC0** (K_T = $4{\cdot}10^{13}$) (**b**) **GC1** (K_T = $3{\cdot}10^{12}$) and (**c**) **GC2** (K_T = $5{\cdot}10^{14}$).

S3. Calculation of Reference G:C Association Constants

As stated above, each macrocycle displayed a markedly different stability, which is clearly noted in the different K_T values: K_T(*c*(**GC2**)$_4$) > 10^{14} > K_T(*c*(**GC0**)$_4$) = $4.3 \cdot 10^{13}$ > K_T(*c*(**GC1**)$_4$) = $3.4 \cdot 10^{12}$ (see Table S1) Since $K_T = EM \cdot K_{G:C}^4$, this difference may come from a different *EM* value, a different $K_{G:C}$ constant, or both. In order to shed some light on the origin of these stability differences, we calculated the reference association constant for each G:C nucleobase pair ($K_{G:C}$) by ^{1}H NMR titrations in THF-d_8. The results are summarized in Figure S3A.

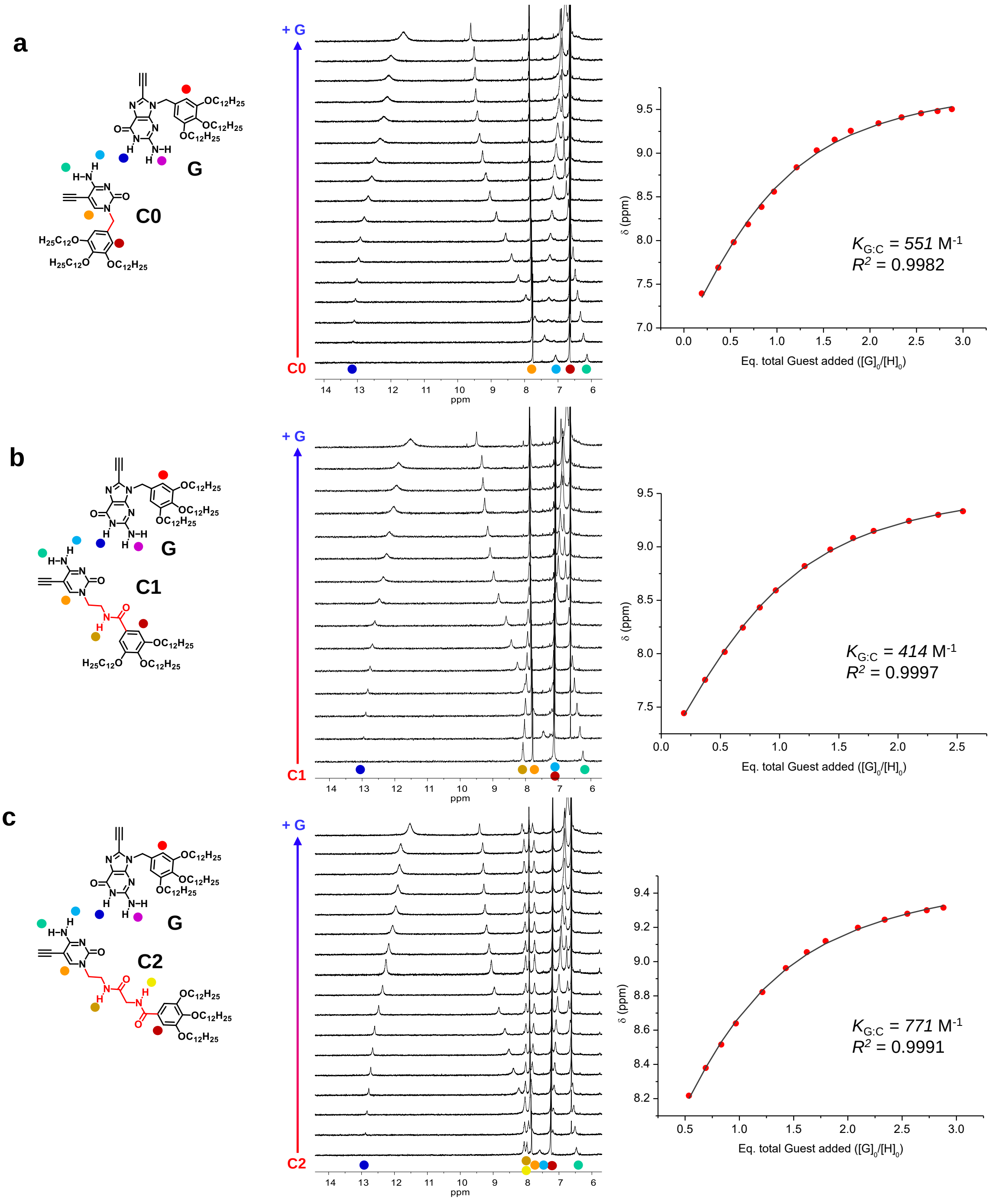

Figure S3A. ^{1}H NMR titration experiments of **G** (guest) over (**a**) **C0**, (**b**) **C1**, or (**c**) **C2** (host) at a constant host concentration of 5 mM in THF-d_8. The corresponding fittings to a 1:1 model are shown at the right.

A sample of the host nucleoside (**C0**, **C1** or **C2**) was dissolved in THF-d_8 at a 5 mM concentration. A portion of this solution was used as the host sample, and the remainder was used to dissolve the guest (**G**; 25 mM), so that the host concentration remained constant throughout the titration. The ^{1}H NMR chemical shift data were then fitted by the software *Equilibria*.[13]

The C nucleobases **C1** and **C2** were also subjected to temperature-dependent studies down to 178 K in THF-d_8 so as to see if we could slow down the exchange between intramolecularly H-bonded conformations in each nucleobase (Figures S3Ba,c). However, although a noticeable broadening was seen for the peripheral amide protons of **C2** at low temperatures, both samples precipitated below 218 K and the amide signals (and actually all protons potentially involved in H-bonding interactions) only showed a gradual downfield shift with decreasing temperature (see also Figure S2A). The ^{1}H NMR spectra of **C1** and **C2** were also recorded in $CDCl_3$ (Figures S3Bb,d).

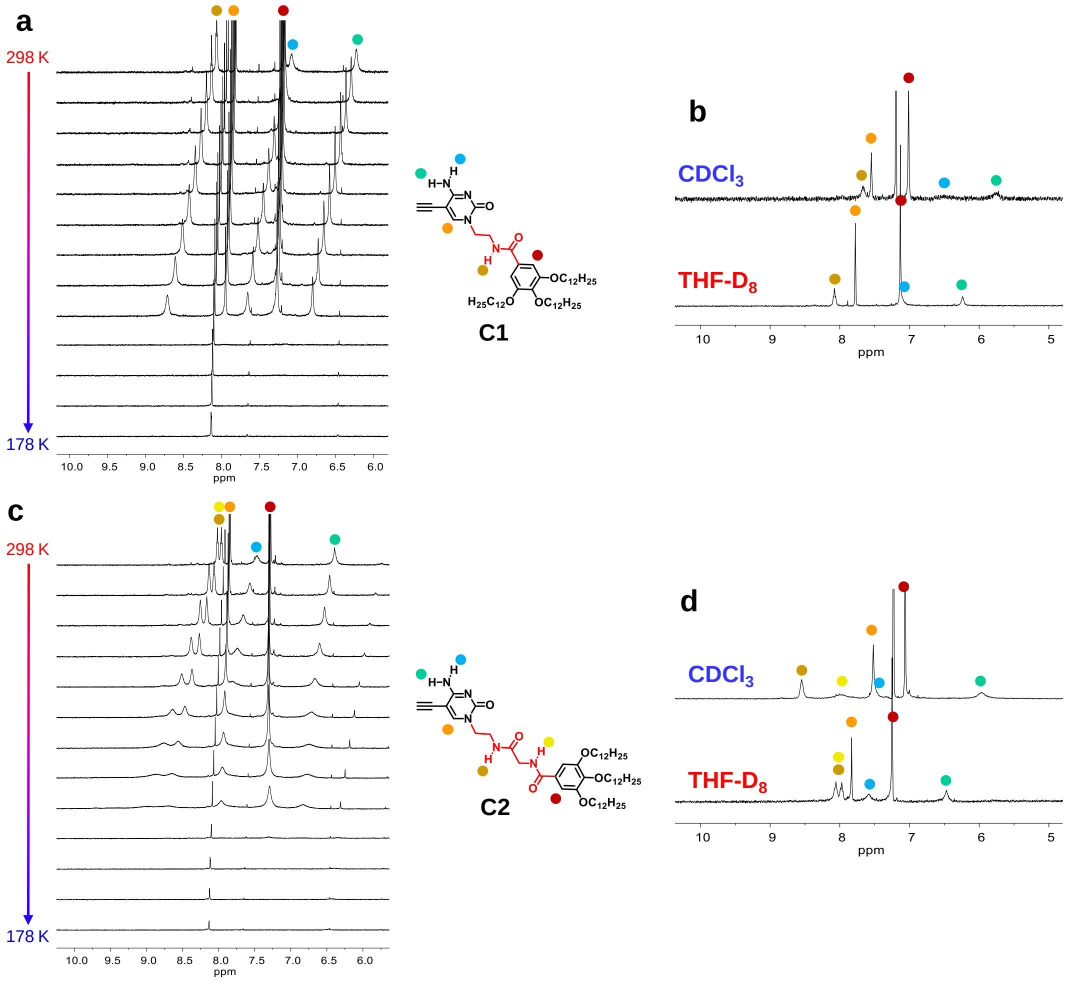

Figure S3B. (**a,c**) Temperature-dependent ^{1}H NMR experiments in THF-d_8 at a 5 mM concentration and (**b,d**) ^{1}H NMR spectra in $CDCl_3$ or THF-d_8 at 298 K of (**a,b**) **C1** or (**c,d**) **C2**.

S4. Cyclic Tetramer-Polymer Equilibria Studied in Apolar Aliphatic Solvents

In order to force further aggregation, we moved to highly apolar alkane media, like methylcyclohexane (MCH), heptane or dodecane. **GC0** is sufficiently soluble in these solvents, but **GC1** and **GC2** or **GC2*** are not, even at low concentrations and/or high temperatures, which already indicated that these compounds form large aggregates that prevented a proper analysis of the polymerization process. In the best case, turbid dispersions are obtained after prolonged heating and sonication that, when analysed spectroscopically, exhibited very different features than those recorded for *c*GC_4 solutions.

We found out that using a small percentage of good solvent (<1% v/v), like $CHCl_3$, toluene or THF, was enough to dissolve these aggregates and to study the supramolecular polymerization process. The transition from the molecularly dissolved *c*GC_4 macrocycles to the strong aggregates formed in alkanes was first studied for all **GC0**-**GC2** compounds by increasing the heptane volume fraction (V_h) in THF-heptane mixtures, as shown in Figure S4A. In order to compare the changes in the two equilibria for the three dinucleosides, the changes at 429 nm *vs* V_h were plotted in Figure S4B.

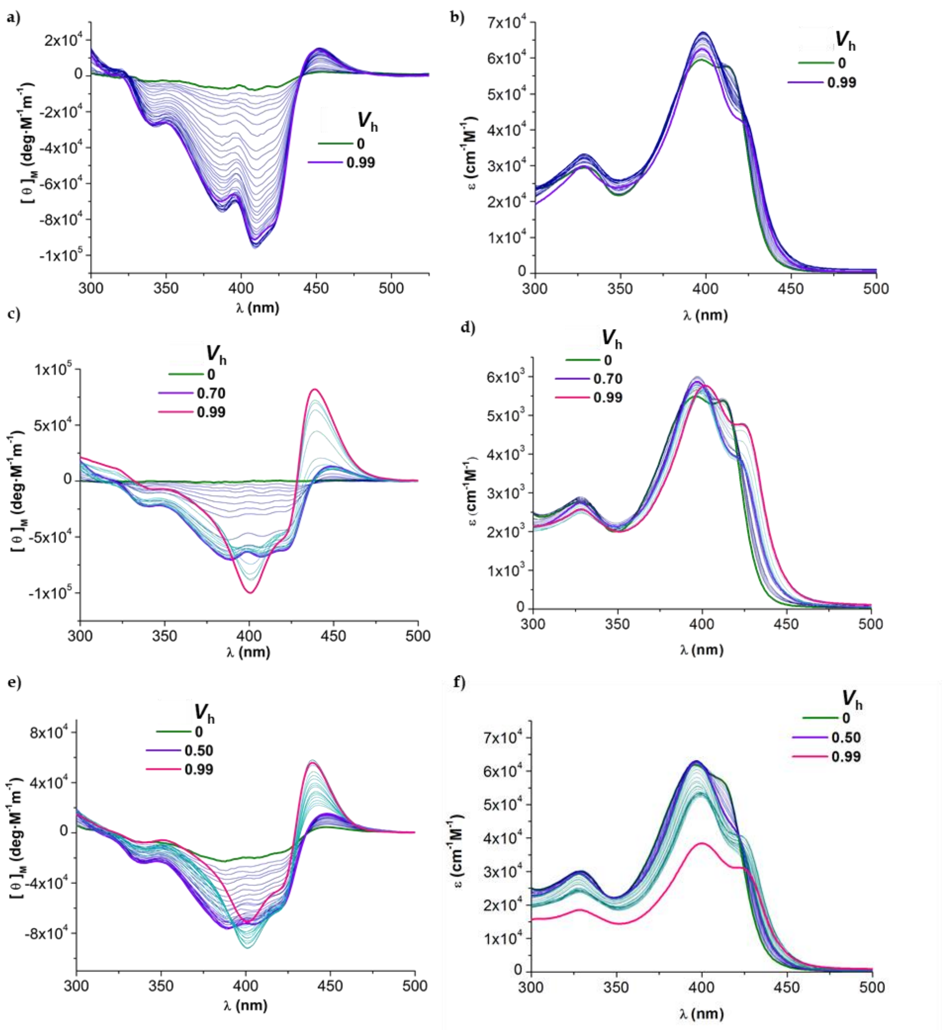

Figure S4A. Titration experiments monitored by (**a,c,e**) CD and (**b,d,f**) absorption of (**a,b**) **GC0**, (**c,d**) **GC1** and (**e,f**) **GC2** in THF at $3.0 \cdot 10^{-5}$ M, with solutions at the same concentration in a 0.01:0.99 THF-heptane solvent mixture. The decrease in CD and absorption intensity of the final stock solution at V_h = 0.99 (pink line in **e,f**) is due to partial precipitation with time.

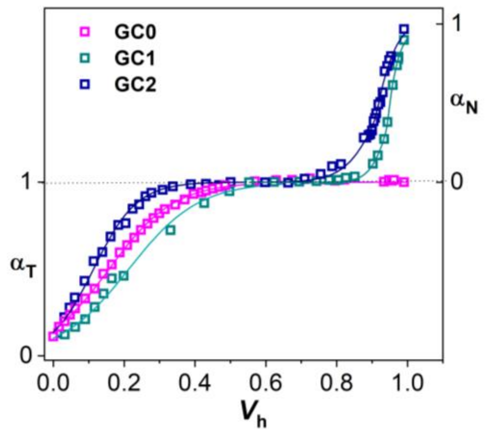

Figure S4B. Normalized CD changes at 429 nm for the titration of **GC0**, **GC1** and **GC2** at $3.0{\cdot}10^{-5}$ M depicted in Figure S4A.

Two transitions are clearly observed: 1) from the monomer (green spectra) to the cyclic tetramer (purple spectra), and then 2) from the cyclic tetramer to tubular polymers (pink spectra). Interestingly, the influence of the peripheral amides was already observed in these experiments. On the one hand, **GC0** did not present the characteristic signal of the nanotubular aggregates, and only the monomer-cyclic tetramer equilibrium was observed during the titration experiment, even up to V_h = 0.99. On the other hand, **GC2** presented the same behaviour as **GC1**. The monomer-macrocycle equilibrium was observed from V_h = 0 to *ca.* 0.4-0.5. Then, a plateau in which the tetramer remained as the most stable species in solution was reached up to V_h = 0.7-0.9. The supramolecular polymerization of the tetramers was then triggered at higher heptane contents. As expected, the polymerization of *c*(**GC2**)$_4$ took place at a lower volume fraction of the bad solvent (V_h = 0.75) than the polymerization of *c*(**GC1**)$_4$. These findings, namely the lack of aggregation of **GC0** and the supramolecular polymerization of **GC2** at a lower V_h, indicated that the presence and number of peripheral amides plays indeed a dominant role in the formation of the polymeric nanotubes.

At the end of these titrations, when reaching high heptane contents, and after some time, which varied from hours to days, scattering becomes very evident for **GC1**, **GC2** and **GC2*** and the samples tended to precipitate, as evidenced spectroscopically by a reduction in absorption intensity.

Since **GC2** was able to form nanotubular aggregates, a study of the mechanism involved in their formation, similar to the one performed in our previous work for **GC1**,[7] was carried out at different concentrations. First, the same kind of experiments as a function of solvent composition were performed at the following concentrations: $1.0{\cdot}10^{-5}$ M, $2.0{\cdot}10^{-5}$ M, and the one already shown at $3.0{\cdot}10^{-5}$ M (Figure S4C). In all cases, the monomer-cyclic tetramer and cyclic tetramer-polymer transitions were observed. As expected, these transitions occurred at slightly lower V_h values as the concentration increased.

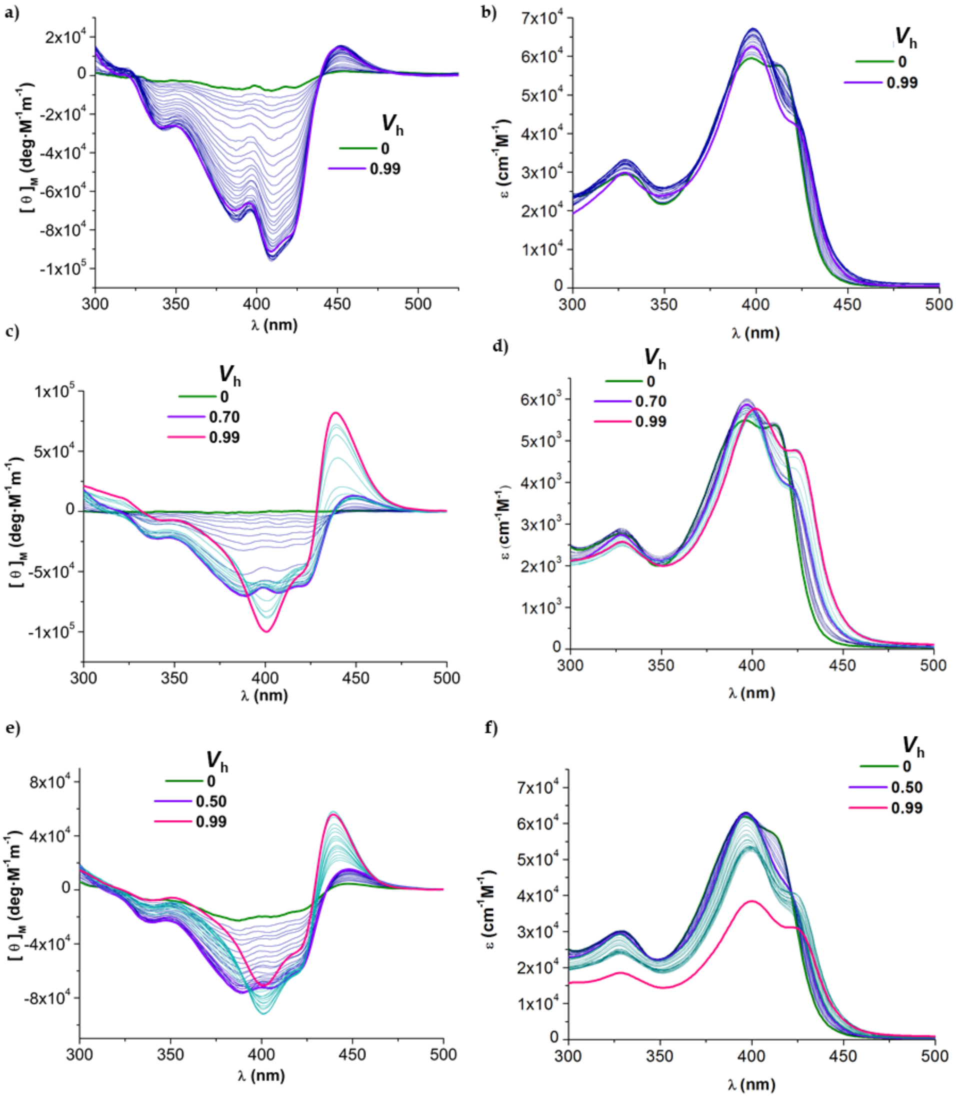

Figure S4C. Titration experiments monitored by CD (**a**,**c**,**e**) and UV-vis (**b**,**d**,**f**) of **GC2** at 1.0·10^{-5} M (**a**,**b**), 2.0·10^{-5} M (**c**,**d**) and 3.0·10^{-5} M (**e**,**f**) in THF with solutions at the same concentration in a 0.01:0.99 THF: heptane solvent mixture. The decrease in CD and absorption intensity of the final stock solution at V_h = 0.99 (pink line in **e** and **f**) is due to partial precipitation with time.

Next, in order to calculate the thermodynamic parameters that govern the supramolecular polymerization process, temperature-dependent experiments were carried out. In these experiments, the cooling rates applied were slow enough (1.0 K·min^{-1}, although we tried slower cooling rates with similar outcome) to try to ensure that the equilibrium is under thermodynamic control and avoid possible kinetic effects. To that end, solutions of **GC2** at different concentrations in a fixed 0.19:0.81 THF-heptane mixture (V_h = 0.81) were heated at 329 K and then slowly cooled down to 270 K while analysing the CD changes (Figure S4D). Even if the small amount of THF present in the mixture did not allow heating the samples above 329 K, complete disruption of the nanotubes was achieved, and only the cyclic tetramer CD signal was observed at high temperatures. As expected, the polymerization process is dependent on concentration, and the melting temperature increases as the sample is concentrated.

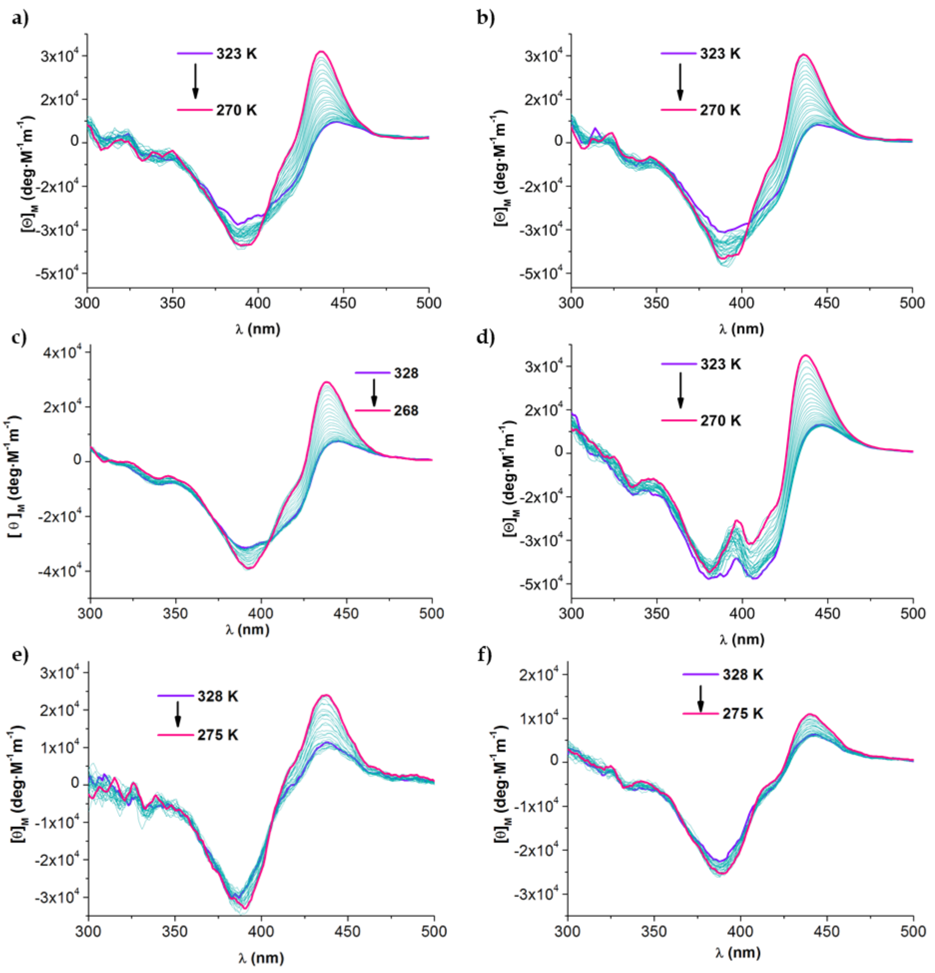

Figure S4D. Temperature-dependent experiments monitored by CD of **GC2** at (**a**) $2.0 \cdot 10^{-5}$ M, (**b**) $2.5 \cdot 10^{-5}$ M, (**c**) $3.0 \cdot 10^{-5}$ M, (**d**) $4.0 \cdot 10^{-5}$ M, (**e**) $1.0 \cdot 10^{-4}$ M and (**f**) $3.0 \cdot 10^{-4}$ M in a 19:81 THF-heptane solvent mixture (V_h = 0.81).

The cooling curves obtained at the different concentrations were then fitted to the isodesmic model (Figure S4E).[14] This model supposes a distribution of oligomeric species with an average degree of polymerization (DP_N) whose value depends on the temperature, the concentration, and the association constant (K). The model considers that the reversible formation of noncovalent bonds is identical for all binding events, implying that the reactivity of the end groups does not change during the supramolecular aggregation process. Thus, the equilibrium constants (K) and Gibbs free energy changes are equal for each step of the growing aggregate. Thus, the degree of polymerization or the molar fraction of aggregated species α_{agg} is related to temperature by means of a sigmoidal relation.

The number-averaged degree of polymerization DP_N can be calculated from α_{agg}:

$$DP_N = \frac{1}{\sqrt{1-\alpha_{agg}}} \qquad (2)$$

Taking into equation:

$$\alpha_{agg} = 1 - \frac{2KC_T + 1 - \sqrt{4KC_T + 1}}{2K^2C_T^2} \qquad (3)$$

This expression can be related to the equilibrium constant K and the total concentration of molecules (C_T) via:

$$DP_N = \frac{1}{2} + \frac{1}{2}\sqrt{4KC_T + 1} = \frac{1 + \sqrt{4KC_T + 1}}{2} \qquad (4)$$

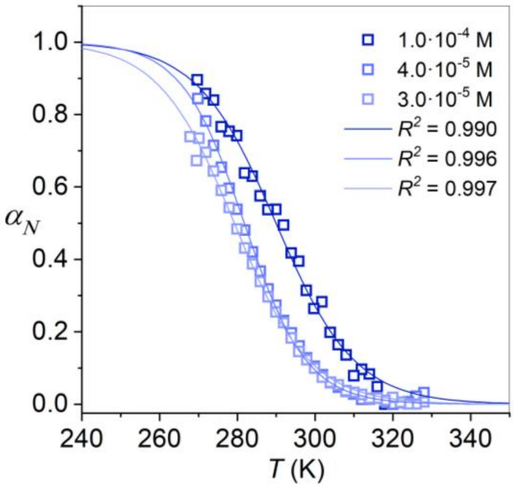

Figure S4E. CD changes at λ = 440 nm of **GC2** in the temperature experiments (Figure S4D) with their corresponding fitting to the isodesmic model.

The most important thermodynamic parameters for the self-assembled processes of **GC1** and **GC2** are shown in Table S2.

Table S2. Thermodynamic parameters calculated from the global fitting of the cooling curves of **GC1** to a nucleation-elongation model[7] and **GC2** to the isodesmic model[14] (Figure S4E).

	**[GC]** (*M*)	**[*c*(GC)$_4$]** (*M*)	T_e [a] (*K*)	K_n [b] (*M^{-1}*)	K_e [c] (*M^{-1}*)	σ [d]	ΔH^0 [e] (*$kJ·mol^{-1}$*)	ΔS^0 [f] (*$J·mol^{-1}·K^{-1}$*)	ΔH_n^0 [g] (*$kJ·mol^{-1}$*)
**GC1**	*2.0·10^{-5}*	*5.0·10^{-6}*	296 ± 1	4.0·10^1	1.3·10^5	3.0·10^{-4}	-116 ± 3	-290 ± 10	-20.1 ± 3.0
	3.0·10^{-5}	*7.5·10^{-6}*	298 ± 1						
			T_m [h] (K)	K [i] (M^{-1})			ΔH^0 [j] ($kJ·mol^{-1}$)	ΔS^0 [j] ($J·mol^{-1}·K^{-1}$)	ΔG^0 [k] ($kJ·mol^{-1}$)
**GC2**	*1.0·10^{-4}*	*2.5·10^{-5}*	290.0 ± 0.3	1.0·10^5		1	-78.6 ± 2.0	-169.0 ± 6.8	-28.3 ± 4.0
	4.0·10^{-5}	*1.0·10^{-5}*	281.8 ± 0.2	0.3·10^5		1	-97.2 ± 2.0	-242.3 ± 6.6	-25.0 ± 4.0
	3.0·10^{-5}	*7.5·10^{-6}*	279.2 ± 0.2	0.4·10^5		1	-82.8 ± 1.8	-191.7 ± 6.0	-25.7 ± 3.5

[a] T_e, elongation temperature. [b] K_n, nucleation constant. [c] K_e, elongation constant. [d] σ, cooperativity factor. [e] ΔH^0, elongation enthalpy [f] ΔS^0, entropy. [g] ΔH^0_n, nucleation enthalpy. [h] T_m, melting temperature. [i] K, isodesmic constant. [j] ΔH^0, ΔS^0 calculated using the Van´t Hoff equation. [f] ΔS^0, calculated using the Gibb's equation. Since the tetramer is considered as the "supramonomer", the total concentration was divided by 4 (2nd column).

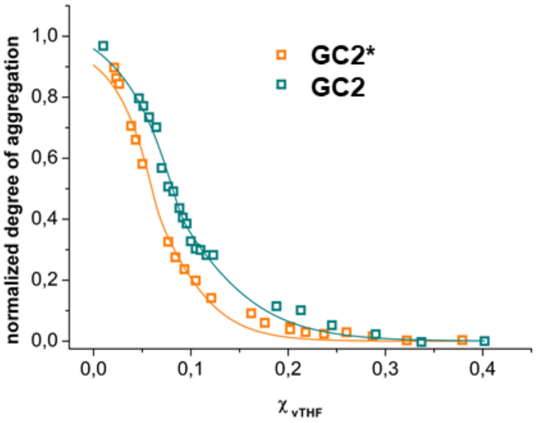

Figure S4F. Comparison of the degree of aggregation of **GC2** and **GC2*** monitored by absorption spectroscopy as a function of the volume fraction of THF in mixtures with heptane. $C = 2.0 \cdot 10^{-5}$ M, $T = 298$ K.

S5. Characterization of the Self-assembled Nanotubes by TEM

We then proceeded to characterize the final **GC1** and **GC2** aggregates obtained after the polymerization process, in order to confirm their dimensions and their tubular nature. TEM proved to be the best method to image the assemblies at smaller scales and measure their individual dimensions. TEM measurements were performed with a JEOL JEM 1400 PLUS equipment working at an accelerating voltage of 40 to 120 kV, which provided higher contrast and resolution. The analysis of **GC1** was performed in our previous work,[7] but some images are reproduced herein for the sake of comparison (Figures S5a-c). Approximately 10^{-5} M solutions of **GC2** at V_h = 0.9 were drop-casted into 200-mesh formvar copper grids coated with carbon (Figures S5d-f).

A general conclusion from all experiments performed with these dinucleobase compounds is that, the longer the time in solution before deposition onto the substrate, the higher the degree of nanotube bundling (please, compare Figures S5a,b,d,e with Figures S5c,f). As a matter of fact, when the dinucleobase compounds were left for several days in solutions of high heptane content, a precipitate emerged, especially in concentrated solutions. Bundling was beneficial for a successful detection of the nanotubes onto the grid, but detrimental for the study of isolated nanotubes. It is important to note that in these experiments the samples were not stained and, as a consequence of the absence of high electron-density atoms in the nanotubes, the presence of several nanotubes in regions of high contrast (dark background) was required in order to observe them clearly.

Mean diameters of about 3.9 nm and 4.0 nm was calculated from the analysis of several TEM images from different samples and regions for **GC1** and **GC2**, respectively. The expected diameter of the (c**GC**$_4$)$_n$ nanotubes were calculated from computational models and 3 different ranges are given in Figure S5g-h, since the cyclic tetramer is not perfectly circular. The inner pore presents a diameter of *ca.* 2.0-2.3, calculated as the longer and shorter distance between π-conjugated walls. The diameter of the external π-conjugated rigid section of the tubes, including the benzylic wedges, oscillates in between 3.5 and 4.7 nm. Finally, the total diameter, including the peripheral alkyl chains in their extended conformation can reach up to 7.8 nm. Hence, we presume the lighter nanotube sections measured by TEM correspond to the hard aromatic section of the cyclic tetramers, while the darker regions between tubes consist of interdigitated peripheral chains.

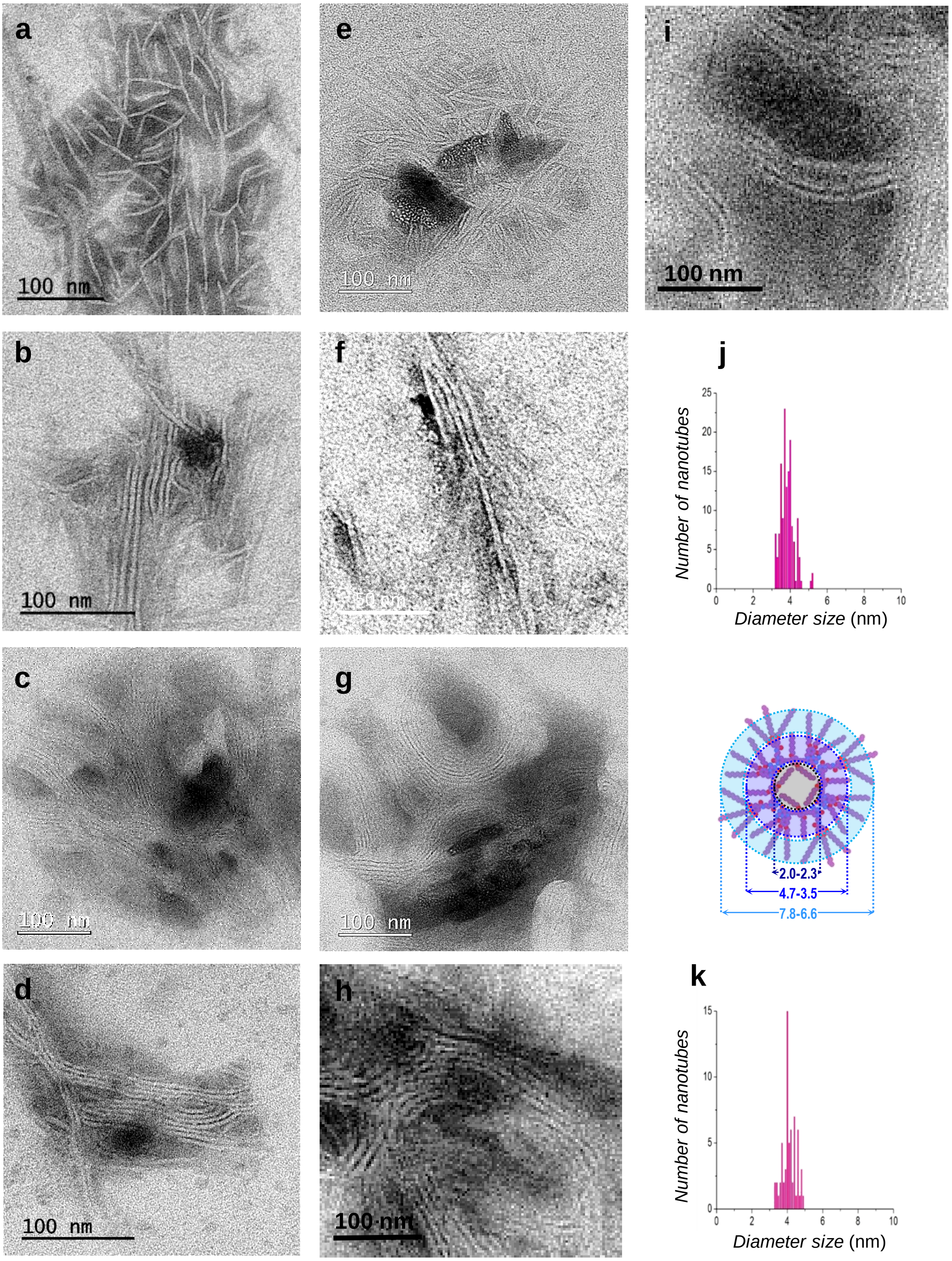

Figure S5. TEM images of (**a-d**) **GC1**, (**e-h**) **GC2** or (**i**) **GC2*** drop-casted from 0.10:0.90 THF-heptane solutions. (**g,h**) Diagrams showing the diameter distribution of the nanotubes for (**j**) **GC1** and (**k**) **GC2**, and model of the section of the nanotubes, indicating the size of the inner pore, the π-conjugated rigid section, and the maximum diameter, including aliphatic chains, of the nanotubes.

S6. References

[1] P. Iqbal, M. Mayanditheuar, L. J. Childs, M. J. Hannon, N. Spencer, P. R. Ashton, J. A. Preece, *Materials* **2009**, *2*, 146-168.
[2] L.-C. Lee, Y. Zhao, *J. Am. Chem. Soc.* **2014**, *136*, 5579-5582.
[3] X.-L. Zheng, R.-R. Tao, R.-R. Gu, W.-Z. Wang, D.-H. Qu, *Beilstein J. Org. Chem.* **2018**, *14*, 2074-2081.
[4] N. Bilbao, V. Vázquez-González, M. T. Aranda, D. González-Rodríguez, *Eur. J. Org. Chem.* **2015**, *2015*, 7160-7175.
[5] A. Ajayaghosh, R. Varghese, S. Mahesh, V. K. Praveen, *Angew. Chem. Int. Ed.* **2006**, *45*, 7729-7732.
[6] C. Weder, M. S. Wrighton, *Macromolecules* **1996**, *29*, 5157-5165.
[7] V. Vázquez-González, M. J. Mayoral, R. Chamorro, M. M. R. M. Hendrix, I. K. Voets, D. González-Rodríguez, *J. Am. Chem. Soc.* **2019**, *141*, 16432-16438.
[8] C. Montoro-García, J. Camacho-García, A. M. López-Pérez, N. Bilbao, S. Romero-Pérez, M. J. Mayoral, D. González-Rodríguez, *Angew. Chem. Int. Ed.* **2015**, *54*, 6780-6784.
[9] M. J. Mayoral, D. Serrano-Molina, J. Camacho-García, E. Magdalena-Estirado, M. Blanco-Lomas, E. Fadaei, D. González-Rodríguez, *Chem. Sci.* **2018**, *9*, 7809-7821.
[10] a) J. Camacho-Garcia, C. Montoro-Garcia, A. M. Lopez-Perez, N. Bilbao, S. Romero-Perez, D. Gonzalez-Rodriguez, *Org. Biomol. Chem.* **2015**, *13*, 4506-4513; b) M. J. Mayoral, J. Camacho-Garcia, E. Magdalena-Estirado, M. Blanco-Lomas, E. Fadaei, C. Montoro-Garcia, D. Serrano-Molina, D. Gonzalez-Rodriguez, *Org. Biomol. Chem.* **2017**, *15*, 7558-7565.
[11] F. Aparicio, M. J. Mayoral, C. Montoro-García, D. González-Rodríguez, *Chem. Commun.* **2019**, *55*, 7277-7299.
[12] Hyss (Hyperquad Simulation and Speciation) program, developed by http://www.hyperquad.co.uk/index.htm.
[13] The Equilibria program was developed by Christopher Marjo, Mark Wainwright Analytical Centre, University of New South Wales, Sydney, Australia. http://www.sseau.unsw.edu.au/.
[14] a) M. J. Mayoral, C. Rest, J. Schellheimer, V. Stepanenko, G. Fernández, *Chem. Eur. J.* **2012**, *18*, 15607-15611; b) M. M. J. Smulders, M. M. L. Nieuwenhuizen, T. F. A. de Greef, P. van der Schoot, A. P. H. J. Schenning, E. W. Meijer, *Chem. Eur. J.* **2010**, *16*, 362-367; c) T. F. A. De Greef, M. M. J. Smulders, M. Wolffs, A. P. H. J. Schenning, R. P. Sijbesma, E. W. Meijer, *Chem. Rev.* **2009**, *109*, 5687-5754.
